# Preoperative chemotherapy response and survival in patients with colorectal cancer peritoneal metastases

**DOI:** 10.1002/jso.27776

**Published:** 2024-07-16

**Authors:** Nadina Tinsley, Sarah T. O'Dwyer, Raghavendar Nagaraju, Bipasha Chakrabarty, Michael Braun, Saifee Mullamitha, Konstantinos Kamposioras, F. E. Marti Marti, Mark Saunders, Hamish Clouston, Chelliah Selvasekar, Jonathan Wild, Malcolm Wilson, Andrew Renehan, Omer Aziz, Jorge Barriuso

**Affiliations:** ^1^ Division of Cancer Sciences, Faculty of Biology, Medicine and Health University of Manchester Manchester UK; ^2^ Christie Peritoneal Oncology Centre (CPOC) at The Christie NHS Foundation Trust Manchester UK

**Keywords:** chemotherapy response, colorectal cancer peritoneal metastases

## Abstract

Treatment guidelines provided by PRODIGE‐7 recommend perioperative systemic chemotherapy before cytoreductive surgery (CRS) for colorectal cancer peritoneal metastases (CRPM). Toxicity with multimodal treatment needs to be better defined. Chemotherapy response and impact on survival have not been reported. We assessed CRPM patients who received systemic oxaliplatin/irinotecan before CRS (preoperative) with Mitomycin C (35 mg/m^2^, 90 min) or Oxaliplatin (368 mg/m^2^, 30 min) heated intraperitoneal chemotherapy (HIPEC). Secondary analysis was performed from a prospective database. Overall survival (OS) in chemotherapy responders (R) and nonresponders (NR) was compared. Toxicity was assessed by rate of adverse events (AEs). From April 2005 to April 2021, 436 patients underwent CRS + HIPEC; 125 (29%) received preoperative chemotherapy. The 112 (90%) received oxaliplatin (64, 57%) or irinotecan (48, 43%). R, defined as complete (CR) or partial response on preoperative imaging and/or postoperative histology, was seen in 71, 63% (53.8–72.3); 16, 14% (8.4–22.2) had CR. Median OS in R versus NR was 43.7 months (37.9–49.4) versus 23.9 (16.3–31.4) *p* = 0.007, HR 0.51 (0.31–0.84). OS multivariable analysis showed HR 0.48 (0.25‐0.95), *p* = 0.03 for chemotherapy response corrected by peritoneal cancer index, completeness of cytoreduction score. CRS led to 21% grade 3–4 AEs versus 4% for preoperative chemotherapy. HIPEC grade 3‐4 AEs were 0.5%. Preoperative chemotherapy response is an independent predictor for OS in CRPM.

## Highlights

- Perioperative chemotherapy is used in the multimodal treatment of colorectal cancer peritoneal metastases (CRPM).
- Preoperative chemotherapy response is an independent predictor for overall survival in patients with CRPM.
- Grade 3–4 adverse events related to mitomycin C (35 mg/m^2^) and Oxaliplatin (368 mg/m^2^) heated intraperitoneal chemotherapy are rare (0.5%).

## 1 INTRODUCTION

Before the PRODIGE‐7 trial, treatment of operable colorectal cancer peritoneal metastases (CRPM) included heated intraperitoneal chemotherapy (HIPEC) with oxaliplatin or mitomycin C (MMC) as an adjunct to cytoreductive surgery (CRS).^1^, ^2^, ^3^ Systemic chemotherapy was reserved for patients with inoperable disease, and these patients demonstrate an inferior survival compared to patients with metastases outside the peritoneal cavity.^4^, ^5^, ^6^, ^7^

In recent clinical practice, patients with borderline resectable disease and those who demonstrate poor prognostic features (high T stage, node involvement, poorly differentiated or signet ring histology) are commenced on chemotherapy with neoadjuvant intent, aiming to downsize CRPM and facilitate conversion to complete CRS. Equally, some unresectable patients who commenced treatment with palliative intent and achieved an adequate response to chemotherapy would go on to have CRS.

Perioperative chemotherapy therefore plays an essential role in the multimodal treatment of operable CRPM and is recommended by international guidelines (ASCO, ESMO).^8^, ^9^ The key goals of preoperative chemotherapy in CRPM are to downsize peritoneal tumour volume, making patients amenable to complete CRS (conversion therapy) and to target local and distant micro‐metastastic disease, decreasing the risk of recurrence (neoadjuvant). This approach with oxaliplatin perioperative chemotherapy led to a median recurrence‐free survival (RFS) of 11.1 months (95% CI 9.0–12.7) and an overall survival (OS) of 41.2 months (95% CI 35.1–49.6) in the control arm of PRODIGE‐7 without additional oxaliplatin HIPEC.^1^ Of note, relatively high complication rates were observed in both arms, notably 32% adverse events (AEs) of grade 3 or higher at 30 days and 15% at 60 days in the systemic chemotherapy + CRS alone arm.^1^

To date, the majority of trials have used oxaliplatin as the systemic chemotherapy agent of choice before or after CRS. PROPHYLOCHIP‐PRODIGE15, COLOPEC and HIPEC T4 were adjuvant HIPEC trials aimed to reduce the risk of CRPM in high‐risk colorectal cancer patients.^10^, ^11^, ^12^ All patients received adjuvant oxaliplatin containing chemotherapy after resection of the high‐risk primary tumour. For peritoneal metastatic disease, PRODIGE‐7 perioperative chemotherapy regimens were also oxaliplatin based.^1^ Finally, CAIRO‐6 comprised multiple perioperative chemotherapy regimens with Bevacizumab, an antibody treatment targeted against vascular endothelial growth factor (VEGF) as a way to include all eligible patients and demonstrate safety and feasibility in phase II.^13^ Although trials have led to changes in current practice, it is important to recognise that these studies are heterogeneous and the optimal regimen is not defined. In addition, systemic chemotherapy response before CRS and its impact on survival for patients with CRPM has not been studied in randomised controlled trials (RCTs). Do date, few studies have reported an OS benefit for CRPM patients to achieve chemotherapy response before CRS. An early study by Passot et al identified 5‐year survival rates were 75% for CRPM patients with complete pathological response to chemotherapy and 57% for those with major responses.^14^ Sugarbaker et al. also showed that complete or near complete response (CR) to neoadjuvant chemotherapy indicated improved survival in patients with CRPM.^15^ The clinical and histopathologic features of 35 patients who achieved long‐term survival also included a complete or near‐CR to preoperative chemotherapy (HR 0.251 [95% CI, 0.092–0.684]; *p* = 0.007.^16^ Finally, chemotherapy responses were recently studied in 30 patients.^17^ A histological response (by tumour regression grade [TRG] and peritoneal regression grade score [PRGS]) to preoperative chemotherapy was associated with longer postoperative OS (mean OS for PRGS 1–2 [74.19 months] vs. PRGS 3–4 [25.27 months] *p* = 0.045, TRG 1–2 [74.58 months] versus TRG 4–5 [25.27 months] *p* = 0.032) and PFS (mean PFS for PRGS 1–2 [58.03 months] vs. PRGS 3–4 [11.67 months], *p* = 0.002, TRG 1–2 [61.68 months] versus TRG 4–5 [11.67 months], *p* = 0.003).

In addition, patients who receive systemic chemotherapy before surgery may have pre‐existing toxicities which could make them more susceptible to AEs related to HIPEC. More emphasis should be directed toward reporting toxicity attributed to perioperative chemotherapy and how this can affect morbidity and OS.

The aim of this study is to evaluate perioperative chemotherapy outcomes in CRPM patients undergoing CRS + HIPEC. The primary objective is to evaluate the impact of preoperative chemotherapy response on patient survival. Secondary and exploratory objectives are to compare outcomes of different preoperative chemotherapy agents, assess toxicity related to receiving preoperative chemotherapy, HIPEC, surgical morbidity and explore chemotherapy intent before surgery.

## 2 MATERIALS AND METHODS

UK regulatory guidelines for audit and research were followed to conduct this study. Permission was granted by the Quality Improvement and Clinical Audit committee at the Christie Hospital NHS Foundation Trust on 12 September 2022, with the project approved as service evaluation (ref 3403).

Inclusion criteria included CRPM patients who had one line of preoperative chemotherapy with oxaliplatin (85 mg/m^2^) or irinotecan (180 mg/m^2^) before CRS + HIPEC from April 2005 to April 2021. Patients were identified from a prospective database held by the Christie Peritoneal Oncology Centre (CPOC) for clinical audit and governance.

Retrospective patient data was collected from electronic patient records. Clinico‐pathologic variables included patient sex, age, primary colorectal tumour site, TNM stage, histology, CRS details (peritoneal cancer index [PCI], completeness of cytoreduction score [CC score], HIPEC agent), molecular profile (RAS, BRAFV600E, PIK3CA, microsatellite status). Preoperative chemotherapy (agent, number of cycles, use of monoclonal antibody [MOAB]) and response to chemotherapy were collected. Chemotherapy response (R) was defined as response seen on imaging with CT Thorax Abdomen Pelvis (CT TAP) confirmed at central multidisciplinary meeting with dedicated peritoneal tumour radiologists, or evidence of tumour regression (TRG 2–4) on postoperative histology^18^; no response (NR) was defined as stable or progressive disease. CR was defined as complete tumour regression (TRG 1) or no evidence of viable tumour on postoperative histology.^18^ AEs were collected and graded according to the Common Terminology Criteria for Adverse Events version 5 (CTCAE V5).^19^ AE causality was attributed to chemotherapy, HIPEC, surgery, or medical complications as stated in clinical notes. Rates of AE were calculated as percentages from the total number of AEs. Patient status (alive/dead or recurrence) and date of last follow‐up for censored cases was collected and analysed. OS was calculated from the time of CRS + HIPEC to death. Patients were followed up until 1 July 2022.

We sought to analyse preoperative chemotherapy outcomes in CRPM patients undergoing CRS + HIPEC. The primary end point of the study was chemotherapy response and impact on OS. Secondary endpoints included OS by chemotherapy agent and the rate of grade 3–4 AEs related to chemotherapy, HIPEC and surgery.

### 2.1 Statistical analysis

Eligible patients who had 1 or more CRS + HIPEC procedures for CRPM and had preoperative chemotherapy before CRS were analysed as distinct entries. Univariable analysis (UVA) for each variable was performed for OS using Kaplan Meier, Log Rank Test and Cox Proportional Hazard model. Only preoperative chemotherapy regimens received before CRS + HIPEC were included in the analysis. Chi square or Fisher's exact test for categorical variables to explore possible associations. Interaction analysis using Cox Proportional Hazard Method for variables showing significant impact on OS by univariate Cox regression. *p*‐Values ≤ 0.05 were considered statistically significant. Variables that demonstrated significant *p*‐values ≤ 0.05 on UVA were selected for multivariable analysis (MVA) using Cox Proportional Hazard Model. Significant interactions were also inputted in the multivariable model. Enter variable selection method was utilised for OS analysis. Analysis was performed using IBM SPSS v28 software and R v4.2.1.

## 3 RESULTS

### 3.1 Patient characteristics

Of 436 CRPM patients who underwent CRS + /− HIPEC from April 2005 to April 2021, 125 (29%) received systemic pre‐CRS chemotherapy; 112 (90%) received preoperative chemotherapy before CRS with either oxaliplatin (64 patients = 57%) or irinotecan (48 patients = 43%) (Figure S1, consort diagram). Patient and chemotherapy characteristics are summarised in Tables 1A and 1B.

**Table 1A:** Characteristics of 112 patients who received preoperative chemotherapy.

Patient characteristics		
Age	Median (range)	61 (21–79)
Sex	Female	60 (54%)
	Male	52 (46%)
Primary site	Left	66 (59%)
	Right	41 (37%)
	Transverse	2 (2%)
Histology	Adenocarcinoma NOS	87 (78%)
	Mucinous	20 (18%)
	Other	5 (4%)
Chemotherapy	Oxaliplatin	64 (57%)
	Irinotecan	48 (43%)
Chemotherapy intent	Neoadjuvant	27 (24%)
	Palliative	85 (76%)
MOAB	None	70 (63%)
	EGFR	29 (26%)
	VEGF	13 (12%)
Response outcomes	Response*	71 (63%)
(%) (95% CI)		(53.8–72.3)
	*Complete response	16 (14%)
		(8.4–22.2%)
	No response	41 (37%)
Operation details	Median PCI (range)	7 (0‐32)
	CC0‐1	96 (86%)
	CC2‐3	14 (13%)
	MMC (HIPEC)	81 (72%)
	Oxaliplatin (HIPEC)	26 (23%)
Molecular profile	RAS mutant	49 (44%)
	BRAFV600E	18 (16%)
	PIK3CA	7 (6%)

*Note*: Characteristics of 112 patients who received pre‐operativepreoperative chemotherapy. Response* signifies a subset of have (*complete response).

Abbreviations: CC, completeness of cytoreduction score; EGFR, epidermal growth factor receptor; HIPEC, hyperthermic intraperitoneal chemotherapy; MMC, mitomycin C; MOAB, monoclonal antibody; NOS, not otherwise specified; PCI, peritoneal cancer index; VEGF, epidermal growth factor receptor; WT, wild type.

**Table 1B:** Characteristics of the 112 patients who received preoperative chemotherapy by chemotherapy agent.

Chemotherapy characteristics							
	Neoadjuvant	Palliative	Cycles	PCI	Response (R)	No response (NR)	Complete response (CR)
Oxaliplatin	23 (36%)	41 (64%)	7*	7.5*	33 (52%)	31 (48%)	8 (7%)
Irinotecan	4 (8%)	44 (92%)	8*	8*	38 (79%)	10 (21%)	8 (7%)

*Note*: Characteristics of the 112 patients who received preoperative chemotherapy by chemotherapy agent.

Abbreviations: CR, complete response; NR, no response; PCI, peritoneal cancer index; R, response.

* Refers to median (cycles and PCI).

Median age was 61 (21–79) and there was a female predominance with 60 patients (54%). More left‐sided colonic tumours were observed, 66 patients (59%). Most, 87 (78%) were adenocarcinoma not otherwise specified (NOS). Mucinous adenocarcinoma defined as ≥50% mucin content as per WHO criteria occurred in 20 patients (18%).^20^

CC scores of 0–1 were achieved in 96 patients (86%). Molecular profile demonstrated 49 patients (44%) were RAS mutant (KRAS, NRAS) and 18 patients (16%) had a BRAFV600E mutation.

The median PCI in the oxaliplatin and irinotecan preoperative chemotherapy groups was comparable, 7.5 and 8, respectively. The median number of chemotherapy cycles received was 7 in the oxaliplatin group and 8 in the irinotecan group. Response (R), comprising complete (CR) or partial response was seen in 71 patients, 63% (95% CI 62.9–63.1). Of these, 16 patients (14%, 95% CI 13.9–14.1) had CR. The rate of CR among oxaliplatin and irinotecan groups was equal, i.e., eight patients (7%) in each group.

Monoclonal antibodies (MOABs) were used in addition to chemotherapy in 42 patients (38%): 29 patients (26%) received an epidermal growth factor receptor (EGFR) antibody and 13 patients (12%) received a VEGF antibody.

Less than a quarter of patients, 27 (24%) commenced chemotherapy with neoadjuvant intent. The majority of patients, 85 (76%) received chemotherapy with palliative intent and subsequent conversion to CRS due to response. The irinotecan group comprised of 48 patients, of which 44 (92%) received treatment with palliative intent; oxaliplatin comprised of 64 patients of which 41 (64%) received treatment with palliative intent.

### 3.2 Outcomes

Median follow up was 28 months (IQR 16–47 months). During this time, a high proportion had disease recurrence, 92 patients (82%); of these, 46 (41%) had peritoneal recurrence, 24 (21%) recurred systemically and 22 (20%) had both peritoneal and systemic (multisite) recurrence. 64 patients (57%) died due to progressive disease. 20 patients (18%) were alive and disease‐free and 28 patients (25%) were alive with disease at the time of this study. OS did not differ significantly between sites of recurrence, *p* = 0.82 (median OS after peritoneal recurrence vs. systemic versus multisite = 29.5 months [23.4–46.4] vs. 30.1 [22.2–not reached, NA] HR 1.10 [0.61–1.99] vs. 30.3 [23.0–NA], HR 1.24 [0.71–2.17], respectively), (Figure S2).

**Figure 1:**
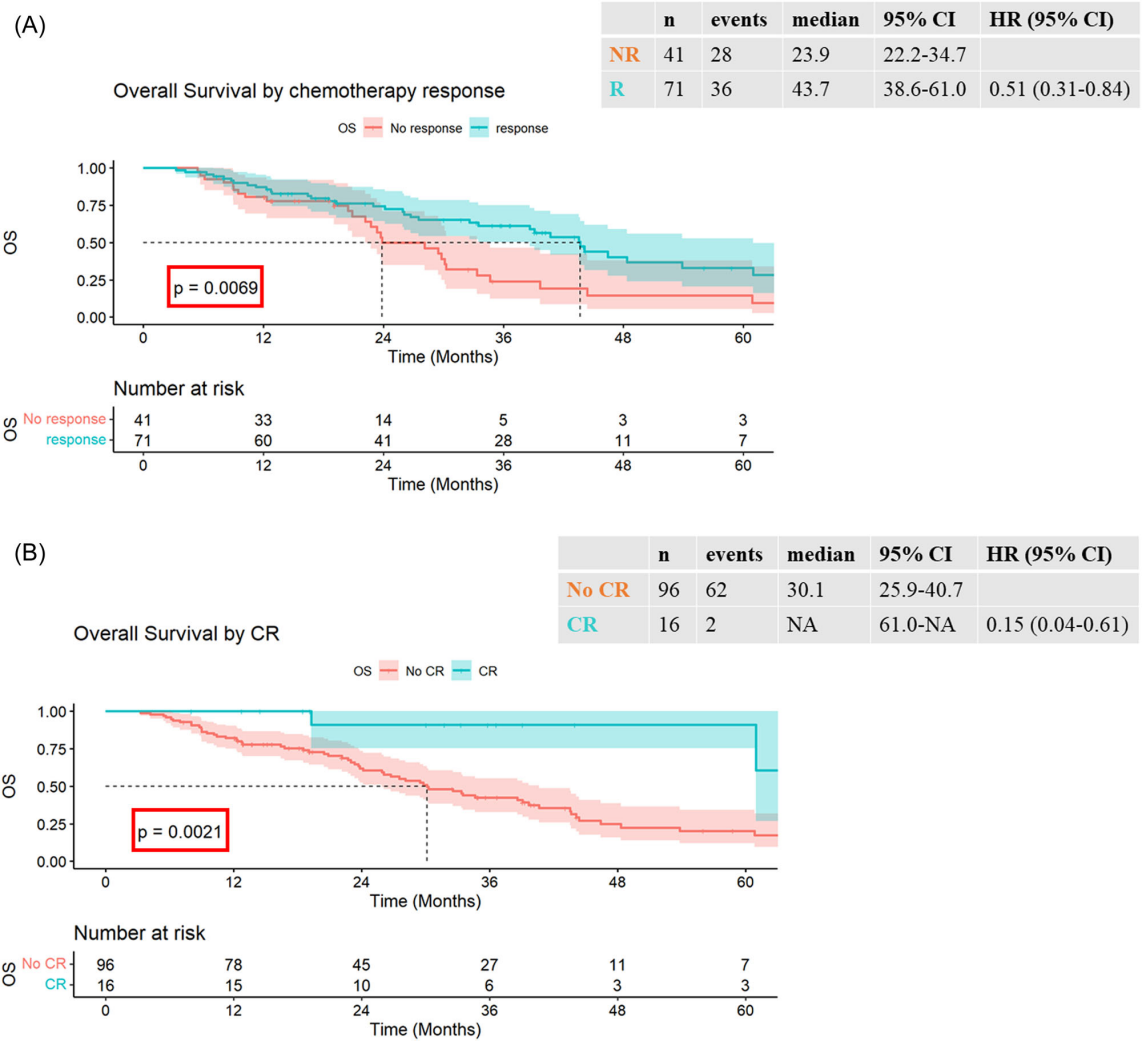
(A) Overall Survival (OS) (months) by perioperative chemotherapy response survival outcome based on patients' response to perioperative chemotherapy. Kaplan Meier survival was plotted for patients who had peri‐operative chemotherapy before CRS + HIPEC. This result has not been adjusted for other clinical factors. (B) OS (months) by perioperative chemotherapy completeness of response Survival outcome based on patients' complete response to perioperative chemotherapy. Kaplan Meier survival was plotted for patients who had perioperative chemotherapy before CRS + HIPEC. This result has not been adjusted for other clinical factors. CI, confidence interval; CR, complete response; CRS cytoreductive surgery; HIPEC, heated intraperitoneal chemotherapy; HR, hazard ratio; NA, not reached; NR, no response; R, response.

For OS, variables with significance on UVA included PCI, *p* = 0.0001 (first comparator PCI < 11 median OS 43.7 [38.6–70.2] vs. PCI 11–15 median OS 29.5 [23.4–not reached, NA], HR 2.34 [1.16–4.73] *p* = 0.018 vs. PCI > 15 median OS 12.3 [9.0–23.7], HR 7.51 [3.88–14.51] *p* < 0.001), CC score, *p* < 0.0001 (first comparator CC0 median OS 43.6 [38.6–60.9] vs. CC1 median OS 23.7 [8–NA], HR 3.24 [1.43–7.34] *p* = 0.005 vs. CC2 median OS 12.3 [9.0–NA], HR 7.95 [3.77–16.76] *p* < 0.001 vs. CC3 median OS 7.2 (3.3–NA), HR 38.05 (11.17–129.61) *p* < 0.001) and chemotherapy response *p* = 0.008 (first comparator no response (NR) median OS 23.9 [22.2–34.7] vs. response (R) median OS 43.7 [38.6–61.0], HR 0.51 [0.31–0.84]) (Table 2, Figure 1A). Patients who achieved CR have not reached median OS (61–NA) compared to non‐CR 30.1 months (25.9–40.7), HR 0.15 (0.04–0.61), *p* = 0.002 (Figure 1B).

**Table 2:** Univariable analysis of the association between clinical factors and overall survival.

	Univariable analysis for OS (*n* = 112)							
Variable	*N*	Events	Median	95% CI	Log‐rank *p* value	HR	95% CI	Cox regression *p* value
**PCI**					**≤0.001**			**<0.001**
PCI < 11 (main comparator)	73	33	43.7	38.6–70.2				
PCI11‐15	16	11	29.5	23.4–NA		2.34	1.16–4.73	**0.018**
PCI > 15	20	17	12.3	9–23.7		7.51	3.88–14.51	**<0.001**
**CC score**					**<0.001**			
CC0 (main comparator)	87	41	43.6	38.6–60.9				
CC1	9	7	23.7	8.02–NA		3.24	1.43–7.34	**0.005**
CC2	10	10	12.3	8.97–NA		7.95	3.77–16.76	**<0.001**
CC3	4	4	7.2	3.29–NA		38.1	11.17–129.61	**<0.001**
T stage					0.31			0.61
T1‐2 (main comparator)	2	0	NA	NA				
T3	22	15	43.6	27.7–NA		1.04	0.14–7.86	0.97
T4	86	47	34.7	26.7–44.1		0.79	0.11–5.78	0.82
Nodal stage					0.41			0.28
N0 (main comparator)	26	12	40.7	29.8–NA				
N1	44	26	28.1	22.2–43.7		1.18	0.54–2.54	0.68
N2	41	25	39.7	26.7–69.8		1.67	0.79–3.52	0.18
Site					0.71			0.71
Right colon	41	20	34.7	23.9‐–NA				
Transverse NOS	3	3	24.1	12.8‐NA		1.59	0.47–5.40	0.46
Left colon	66	40	38.6	27.5–46.4		1.17	0.68–2.01	0.57
Histology					0.73			0.76
Adeno NOS	87	49	38.6	28.1–44.1				
Mucinous	20	11	33.5	16.8–NA		1.15	0.60–2.20	0.68
Signet ring	4	3	41.8	19.1–NA		0.72	0.22‐2.35	0.59
Grade					0.79			0.79
Well (main comparator)	6	3	26.7	16.8–NA				
Moderate	82	48	38.6	29.5–46.4		0.66	0.20–2.16	0.50
Poor	17	10	33.5	24.1–NA		0.66	0.18–2.42	0.53
**Chemotherapy response**					**0.007**			**0.008**
No response (main comparator)	41	28	23.9	22.2–34.7				
Response	71	36	43.7	38.6–61.0		0.51	0.31–0.84	
**Completeness of response**					**0.002**			**0.008**
No CR (main comparator)	96	62	30.1	25.9–40.7				
CR	16	2	NA	61.0–NA		0.15	0.04–0.61	
Chemotherapy agent					0.064			0.066
Oxaliplatin (main comparator)	64	38	28.1	24.1–34.7				
Irinotecan	48	26	44.1	39.7–61.0		0.62	0.37–1.03	
MOAB use					0.51			0.16
No MOAB (main comparator)	70	38	33.4	26.7–43.6				
MOAB	42	26	43.7	24.1–61.0		0.69		0.41‐1.15
MOAB agent					0.75			0.24
No MOAB (main comparator)	70	38	33.4	26.7–43.6				
EGFR ab	29	16	43.7	22.2–NA		0.58	0.31–1.09	0.09
VEGF ab	13	10	46.7	24.1–NA		0.88	0.45–1.74	0.72
HIPEC agent					0.59			0.33
MMC (main comparator)	81	42	26.1–46.4					
Oxaliplatin HIPEC	26	17	33.5–69.8			0.76	0.44–1.32	
HIPEC agent in chemo responders (*n* = 71)					0.82			0.82
MMC (main comparator)	48	21	43.7	26.7–NA				
Oxaliplatin	20	12	44.1	38.6–NA		0.76	0.44–1.32	
Molecular profile								
RAS status					0.92			0.92
RAS WT (main comparator)	62	32	33.4	24.1–69.8				
RAS mutant	49	31	39.1	30.1–53.9		1.03	0.62–1.69	
BRAFV600E Status					0.84			0.84
BRAFV600E WT (main comparator)	93	52	33.4	26.7–44.4				
BRAFV600E mutant	18	11	39.7	32.6–NA		0.94	0.49–1.81	
PIK3CA Status					0.56			0.56
PIK3CA WT (main comparator)	104	58	34.7	28.1–NA				
PIK3CA mutant	7	5	32.6	12.3–NA		1.32	0.53–3.29	

*Note*: Univariable analysis of the association between clinical factors and overall survival. Log‐rank performed in R. Cox proportional hazard analysis performed in SPSS. Variables in bold and underlined had significant *p‐*values < 0.05 and were selected for subsequent multivariable analysis.

Abbreviations: ab, antibody; CC, cytoreductive score; CI, confidence interval; CR, complete response; EGFR, epidermal growth factor receptor; HIPEC, heated intraperitoneal chemotherapy; HR, hazard ratio; MMC, mitomycin C; MOAB, monoclonal antibody; NA not reached; NOS, not otherwise specified; NR, no response; OS, overall survival; PCI, peritoneal cancer index; R, response; VEGF, epidermal growth factor receptor; WT wild type.

There was no statistical difference for OS between HIPEC agents (MMC vs. Oxaliplatin) in the overall population or in subgroup analysis of chemotherapy responders (median OS in chemotherapy responders with MMC 43.7 [26.7–NA) vs. median OS with Oxaliplatin HIPEC 44.1 [38.6–NA], HR 0.76 [0.44–1.32]) *p* = 0.82 (Table 2).

There was a trend to a better OS with irinotecan (median OS 44.1 months [39.7–61.0]) vs. oxaliplatin (median OS 28.1 [24.1–34.7], HR 0.62 [0.37–1.03]), *p* = 0.064. This is explained by a statistically significant correlation between chemotherapy intent and chemotherapy agent by Fisher's exact test *p* < 0.001 (Table S1). In this patient cohort, palliative patients with chemotherapy response are more likely to proceed to CRS + HIPEC. There was a higher proportion of palliative patients on Irinotecan (Tables 1B and 3).

**Table 3:** Multivariable analysis of the association between chemotherapy response and other significant variables with overall survival.

Multivariable analysis (*n* = 112) for OS			
Event 60 (53.6%) Censored 48 (42.9%) Missing 4 (3.6%)			
	HR	95% CI	*p* Val
PCI overall			0.30
PCI 11–15	1.74	0.78–3.85	0.17
PCI > 15	2.09	0.65–6.67	0.22
CC score overall			0.13
CC1	1.18	0.37–3.78	0.78
CC2	2.21	0.69–7.12	0.18
CC3	8.83	1.38–56.38	0.02
Chemo response	0.48	0.25–0.95	0.03
CC score* chemo response			0.07
CC score 1 versus 0* chemo response	2.61	0.42–16.23	0.31
CC score 2 versus 0* chemo response	4.51	0.92–22.04	0.06
CC score 3 versus 0* chemo response	11.91	1.22–116.44	0.03

*Note*: Multivariable analysis of the association between chemotherapy response and other significant variables with overall survival. Multivariable analysis performed in SPSS using Cox proportional hazard method (all variables enter step 1).

Abbreviations: CC, completeness of cytoreduction score; chemo response, chemotherapy response; HR, hazard ratio; LC, lower confidence interval; OS, overall survival; PCI, peritoneal cancer index; p val, p‐value; UCI, upper confidence interval.

OS was not statistically different with the addition of a MOAB *p* = 0.51 (chemotherapy alone median OS 33.4 months [26.7–43.6] vs. chemotherapy + MOAB median OS 43.7 [24.1–61.0], HR 0.69 [0.41–1.15]). No difference was observed by MOAB agent (Table 2).

Cox Proportional Hazard interaction analysis revealed a highly significant statistical interaction for OS between CC score and chemotherapy response, CCscore*chemo response *p* < 0.001. With increasing CC score, the interaction shows high levels of significance (CC1 vs. CC0*chemo response *p* = 0.032, HR 3.62 [1.11–11.76], CC2 vs. CC0*chemo response *p* < 0.001, HR 9.26 [3.02‐28.4], CC3 v sCC0*chemo response *p* < 0.001, HR 87.0 [13.89–542]) (Table S2).

MVA for OS using Cox Proportional Hazard Model inputted all variables which showed significance for OS on UVA (PCI, CC score, chemotherapy response) and significant interactions (CCscore*chemo response). Chemotherapy response shows significance, *p* = 0.032, HR 0.48 (0.25–0.95) and is an independent predictor for OS (Table 3).

### 3.3 Toxicity

Treatment toxicity according to CTCAE V5 was assigned to preoperative chemotherapy, HIPEC, surgery or other medical causes (Table 4). A total of 214 AEs were recorded (172, 80% grade 1–2, 42, 20% grade 3–4). The surgical procedure was responsible for the highest number of grade 1–2 (77, 36%) and 3‐4 (28, 13%) AEs. The most common AE attributed to surgery was anaemia (36% grade 1–2, 10% grade 3–4). There were 75 (35%) grade 1–2 and 9 (4%) grade 3–4 AEs attributed to preoperative chemotherapy. The most common grade 1–2 AE due to preoperative chemotherapy was peripheral neuropathy (8%). Noteworthy grade 3–4 events consisted of neutropaenia (1%), anaemia (1%) and coronary vasospasm (1%). HIPEC toxicity was predominantly due to MMC, with only 1 (0.5%) grade 1–2 AE (thrombocytopaenia) related to Oxaliplatin HIPEC. In general, lower rates of AEs were observed for HIPEC compared to surgery and chemotherapy. There were 13 (6%) grade 1–2 and 1 (0.5%) grade 3–4 AE (thrombocytopenia < 50,000). No peripheral neuropathy was observed due to HIPEC and there was no worsening of existing peripheral neuropathy or other toxicities due to previous chemotherapy use. Across all multi‐modal treatment, no grade 5 AEs were observed.

**Table 4:** Toxicity profile of the 112 patients who received preoperative chemotherapy.

Toxicity profile								
All grade AEs			*n* = 214					
Grade 1–2 AEs			172 (80%)					
Grade 3–4 AEs			42 (20%)					
Chemotherapy toxicity			HIPEC toxicity		Surgical toxicity		Medical toxicity	
*n* = 84 (39%)			*n* = 14 (7%)		*n* = 105 (49%)		*n* = 11 (5%)	
Grade 1–2	Any	75 (35%)	Any	13 (6%)	Any	77 (36%)	Any	7 (3%)
Grade 3–4	Any	9 (4%)	Any	1 (<1%)	Any	28 (13%)	Any	4 (2%)
Grade 1–2	Neuropathy	18 (8%)	Thrombocytopaenia	9 (4%)	Anaemia	76 (36%)	Gastroenteritis/enterocolitis	2 (2%)
	Lethargy	14 (7%)	Neutropaenia	4 (2%)	Ileus	1 (<1%)	Lung infection	2 (2%)
	Neutropaenia	9 (4%)					Device associated infection	1 (<1%)
	Mucositis	9 (4%)					Infection	1 (<1%)
	Diarrhoea	9 (4%)					UTI	1 (<1%)
	Vomiting	5 (2%)						
	PPE	3 (1%)						
	Alopecia	3 (1%)						
	Constipation	3 (1%)						
	Laryngospasm	1 (<1%)						
	Thrombocytopaenia	1 (<1%)						
Grade 3–4	Neutropaenia	2 (1%)	Thrombocytopaenia	1 (<1%)	Anaemia	22 (10%)	Liver abscess	1 (<1%)
	Coronary vasospasm	2 (1%)			Abdominal haemorrhage	2 (1%)	UTI	1 (<1%)
	Anaemia	2 (1%)			Abdominal infection	1 (<1%)	Infection	1 (<1%)
	Vomiting	1 (<1%)			Ileus	1 (<1%)	PE	1 (<1%)
	Constipation	1 (<1%)			Small bowel obstruction	1 (<1%)		
	Lethargy	1 (<1%)			Dehydration	1 (<1%)		

*Note*: Adverse events by multimodal treatment received. Toxicity profile of the 112 patients who received preoperative chemotherapy. Adverse events by multimodal treatment received. % calculated from total number of AEs (*n* = 214).

Abbreviations: AE(s), adverse event(s); HIPEC, heated intraperitoneal chemotherapy; PE pulmonary embolism; PPE, palmoplantar erythema; UTI, urinary tract infection.

## 4 DISCUSSION

This is the first study with a substantial number of patients to report preoperative (neoadjuvant plus conversion) chemotherapy response and its impact on OS in patients with CRPM undergoing CRS + HIPEC. Preoperative chemotherapy response is an independent predictor for OS and patients who respond to chemotherapy have a median OS of 43.7 months. This is comparable to 41.2 months median OS reported in PRODIGE‐7.^1^ In our study, patients who do not respond to chemotherapy have a poor median OS of 23.9 months, similar to OS with systemic chemotherapy in other unresectable settings.^21^

We also report in detail the toxicities attributed to preoperative chemotherapy, HIPEC and surgery. As expected, the surgical procedure is associated with the highest AEs (grade 1–2 36%, grade 3–4 13%). Preoperative chemotherapy was well tolerated with 4% grade 3‐4 AEs (namely myelosuppression, coronary vasospasm, gastrointestinal toxicity). Toxicity related to HIPEC was due to MMC and comprised of grade 1–2 myelosuppression (6% neutropaenia, thrombocytopaenia). In our study, HIPEC was well tolerated and not associated with significant morbidity, despite these patients potentially being at risk of cumulative toxicity due to previous systemic chemotherapy exposure. There was only 1 grade 3–4 AE due to MMC (thrombocytopaenia) and none related to Oxaliplatin HIPEC.

There are clear advantages to administering chemotherapy preoperatively. Firstly, it provides biological information of response and treatment resistance. Biological information of response may guide subsequent treatment, which could be with CRS or continuation of systemic treatment/addition of MOAB, where median OS is recognised to reach 36 months with EGFR antibodies.^21^ It can be used to achieve disease control and downstaging, facilitating surgical planning and conversion to CRS. Chemotherapy given pre‐operatively means no delay in starting chemotherapy after surgery, while allowing recovery of performance status and it potentially targets micro‐metastases responsible for relapses to be treated early. We demonstrate tolerable toxicity which does not result in patient deterioration or an inability to proceed to surgery.

With regard to HIPEC, we report no difference in OS between MMC and Oxaliplatin. In addition, there are no differences in OS between HIPEC agents in chemotherapy responders. It would be difficult to quantify benefit to a HIPEC agent in this patient group. Our chemotherapy responders reached OS comparable to PRODIGE‐7, which suggests the PRODIGE‐7 patient cohort also benefited from preoperative chemotherapy, where additional oxaliplatin HIPEC did not show a benefit.^1^ Conversely, we report few low grade toxicities with our HIPEC agents, of which only 1 grade 1–2 AE was due to oxaliplatin HIPEC at the lower dose of 368 mg/m^2^. This suggests HIPEC with MMC (35 mg/m^2^) and Oxaliplatin (368 mg/m^2^) does not result in significant harm to CRPM patients at CRS.

We adopted a rigorous approach for calculating OS. Each variable was interrogated in turn and potentially confounding variables were analysed for interactions and correlations. Significant variables and interactions were inputted in our multivariable model. PCIs were categorised as per PRODIGE‐7 to allow reproducibility of results.^1^ Our results are comparable in terms of OS to those of the large RCT.^1^ It is worth noting that our study shows that PCI loses significance when CC score is corrected by chemotherapy response (CCscore*chemo response interaction). This highlights the importance of chemotherapy response for OS.

Our results uncover a new avenue to explaining the results of PRODIGE‐7; if we consider that the randomisation process resulted in responders being equally distributed in both arms, we can hypothesise that patients that achieve an objective response to systemic FOLFOX do not get an additive effect from HIPEC with the same agent, as the maximum benefit from this agent has already been achieved. Also, HIPEC schedules with oxaliplatin include systemic 5‐Fluorouracil, based on clinical trials that showed single agent oxaliplatin does not produce objective responses in any setting.^22^ In addition, recent organoid models have shown that single agent oxaliplatin is not active in organoids derived from peritoneal metastasis.^23^ No models test the use of oxaliplatin in combination with 5‐Fluorouracil. Finally, organoid cultures do not include stroma which is likely an important factor in CRPM.^24^, ^25^

Potential limitations of this real‐world outcomes study include its retrospective approach, relying on patient records for data collection. The higher number of recent CRS procedures is reflected in the median follow‐up, despite the study including patients over a period of 16 years. Our sample size is 125 patients, of which 112 received treatment with oxaliplatin or irinotecan and this reflects real‐world practice. We ensured inclusion of all eligible patients at a high‐volume peritoneal tumour centre.

It is important to recognise that patients who receive preoperative chemotherapy may represent a heterogenous group, where chemotherapy intent can be neoadjuvant or palliative and this could influence the treatment received. Treatment with a MOAB is subject to specific UK NHS guidance and funding rules. Anti‐VEGF treatment is used less frequently, as it is only available in the private sector. In addition, difference in outcomes between right and left colon with regard to EGFR inhibitors could not be studied due to small patient numbers.

This surgical cohort reports a higher proportion of left sided colonic tumours, which may have a better prognosis. Data on microsatellite status is missing in historical patients, as MSI status started to be collected prospectively in 2020 at our institution. Interestingly, a relatively high proportion of patients (16%) had a BRAFV600E mutation and this may be reflected in our higher than expected disease recurrence. This small subgroup of patients had comparable outcomes following CRS to wild‐type patients, therefore within the limitations of our study, we believe BRAF mutant tumours should still be considered for CRS.

We have recently demonstrated that outcomes for our chemotherapy naïve cohort are better (median OS 47.2 months) than those who have chemotherapy.^26^ In said study, we show that patients who are given chemotherapy upfront are more likely to have higher PCI and this could affect OS.^26^ Chemotherapy outcomes after CRS (in patients who achieve good CC scores) were not analysed here. However, our other work shows that in 34 patients, postoperative chemotherapy leads to a median OS that has not yet been reached, *p* = 0.03.^26^ There may be advantages to chemotherapy after CRS, where immediately resectable patients can still benefit from chemotherapy and avoid progressive disease if no response is achieved.

In patients who achieve response, there were no large differences between irinotecan and oxaliplatin preoperative chemotherapy and the rate of CR was similar. At our institution, the choice of preoperative agent upfront can influence further treatment, such that patients who receive prior oxaliplatin are preferentially treated with MMC HIPEC, to avoid the risk of hypersensitivity to oxaliplatin or any cumulative neuropathy (see protocols in Appendix 1).

We observed 37% of patients without response to the therapy received, of which a subset of patients had oxaliplatin pre‐operatively. It is possible that these patients had a primary oxaliplatin resistance. Several groups have molecularly characterised aspects of CRPM and found that tumours appear increasingly homogenous and categorise as an aggressive highly angiogenic mesenchymal phenotype (consensus molecular subtype, CMS 4).^24^, ^27^ To date, studies believe this patient group may derive more benefit from irinotecan and consideration of a VEGF inhibitor.^28^

Finally, we demonstrate that multimodal treatment with preoperative chemotherapy, CRS + HIPEC has improved CRPM outcomes for patients such that there are no large differences in survival between metastatic sites after recurrence (Figure S1). In addition to current MDT processes, we propose that chemotherapy response should be crucial in assessing whether patients should proceed to CRS, advise which agents to use and decide on the use of postoperative chemotherapy. It is likely that combinations of agents are required in the treatment of CRPM due to the complexity of this disease. Indeed, there is no randomised trial data for combining multimodal and multiagent treatment with oxaliplatin perioperative chemotherapy and MMC HIPEC or irinotecan perioperatively with Oxaliplatin HIPEC. More understanding is needed into the biology of the disease by undertaking translational research and identifying patients who are more likely to respond to treatment.

## CONFLICT OF INTEREST STATEMENT

All authors will complete the electronic form to report potential conflicts of interest

Dr Jorge Barriuso declares

*Receipt grants, or research support:* PFIZER, IPSEN, NOVARTIS, AAA, Cancer Research UK grant C64263/A29365

*Receipt travel expenses:* PFIZER, IPSEN, NOVARTIS, AAA, Nanostring, RAND SPA

*Receipt honoraria or consultation fees*: SERVIER, PFIZER, IPSEN, NOVARTIS, NUTRICIA, MEDICOVER, RAND SPA

*Nonfinancial conflicts*: Member of Magnitude of Clinical Benefit Scale (MCBS) extended ESMO working group

Professor Omer Aziz declares

*Receipt grants, or research support: Cancer Research UK grant C64263/A29365 Receipt honoraria or consultation fees*: Smart Surgical Appliance Limited

Professor Sarah T O'Dwyer declares

*Cancer Research UK grant C64263/A29365, Receipt travel expenses*: RAND

## SYNOPSIS/IMPLICATIONS FOR PRACTICE

Perioperative chemotherapy is recommended by international Oncology guidelines for the treatment of resectable colorectal cancer peritoneal metastases (CRPM). Unlike other metastatic sites, chemotherapy response before surgery (preoperative) and impact on overall survival has not been systematically evaluated. This study reports preoperative chemotherapy response is an independent predictor for overall survival in patients with CRPM. Patients who do not achieve response should be the main focus of translational research to investigate treatment resistance. We also assessed adverse events (AEs) related to multimodal treatment. Grade 3–4 AEs related to Mitomycin C (35 mg/m^2^) and Oxaliplatin (368 mg/m^2^) heated intraperitoneal chemotherapy are rare (0.5%).

## Supporting information

Supporting information.

Supporting information.

Supporting information.

Supporting information.

Appendix 1: Local HIPEC protocols.

Supporting information.

## Data Availability

All data subject to confidentiality clauses is available from the corresponding author upon request.
