# Supplementary material for: Preoperative chemotherapy response and survival in patients with colorectal cancer peritoneal metastases
Source: J Surg Oncol. 2024 Jul 16;130(6):1422–32. doi: 10.1002/jso.27776 (PMC11826003; doi:10.1002/jso.27776)
Supplement: Supplementary file 1 — Supporting information. [file JSO-130-1422-s003.pdf]

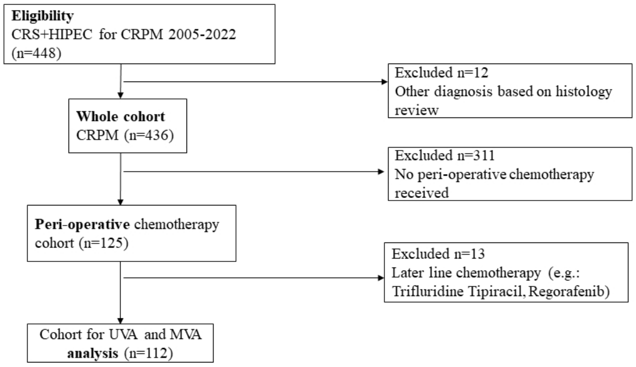

Supplementary figure 1: Patient enrolment CONSORT Flow chart diagram. Patients were identified who had cytoreductive surgery + HIPEC at Christie Hospital for colorectal cancer peritoneal metastases from 2005-2022. Included patients received chemotherapy with oxaliplatin or irinotecan before surgery (later line chemotherapy regimens were excluded). Abbreviations: CRS cytoreductive surgery; HIPEC hyperthermic intraperitoneal chemotherapy; CRPM colorectal cancer peritoneal metastases; UVA univariable analysis, MVA multivariable analysis.

338x190mm (96 x 96 DPI)

	n	events	median	95% CI	HR (95% CI)
Peritoneum	46	32	29.5	23.4-46.4	
Systemic	24	14	30.1	22.2-NA	1.10 (0.61-1.99)
Multi-site	22	18	30.3	23.0-NA	1.24 (0.71-2.17)

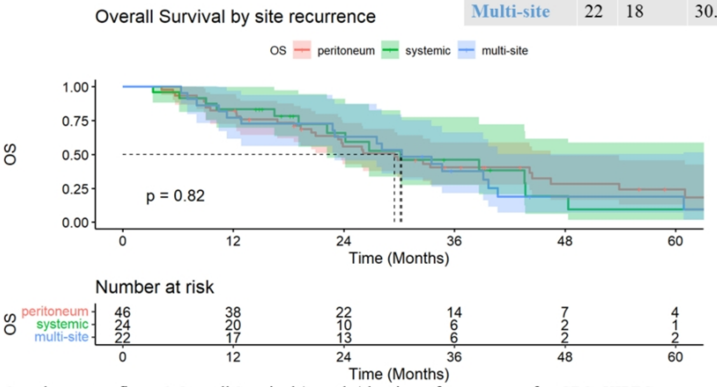

Supplementary figure 2 Overall Survival (months) by sites of recurrence after CRS+HIPEC
Survival outcome based on patient recurrence to peritoneum, systemic or multi-site (peritoneum and systemic). Kaplan Meier survival was plotted for patients who had recurred after CRS+HIPEC. This result has not been adjusted for other clinical factors. Abbreviations: CRS cytoreductive surgery; HIPEC heated intraperitoneal chemotherapy; NA not reached; HR hazard ratio; CI confidence interval

338x190mm (96 x 96 DPI)

Chemo intent * Chemo agent Crosstabulation				
	Oxaliplatin	Irinotecan	Total	p val
Chemo intent (N)	23	4	27	
Chemo intent (P)	41	44	85	
**Total**			112	
**Fisher's exact (2 sided)**				<**0.001**

Supplementary table 1: Crosstabulation of chemotherapy intent and chemotherapy agent. 1 sided Fisher's exact test p value illustrated. Abbreviations: chemo chemotherapy; N neoadjuvant; P palliative; p val p value

338x190mm (96 x 96 DPI)

Variables in analysis			
	HR	95% CI	P val
**CC score * chemo response**			<0.001
**CC score 0 vs 1 * chemo response**	3.62	1.11-11.76	0.032
**CC score 0 vs 2 * chemo response**	9.26	3.02-28.40	<0.001
**CC score 0 vs 3 * chemo response**	86.80	13.89-542.23	<0.001

Supplementary table 2: Cox proportionality interaction analysis by Cox proportional hazard method.
First indicators set as CC0 and NR (no response). Interaction analysis shows CC scores and their interaction with NR vs R (response) for OS. 2 cases with missing values excluded from analysis. Abbreviations: CC score completeness of cytoreduction score; chemo response chemotherapy response; HR hazard ratio; CI confidence interval; p val p value

338x190mm (96 x 96 DPI)

The Christie NHS
NHS Foundation Trust

Document Title:	***Treatment Protocol for Mitomycin Intraoperative Intraperitoneal Chemotherapy (HIPEC)***
Approved by:	Dr Mike Braun- Consultant medical oncologist<br>Peritoneal Service MDT
Version no:	1.1
Date of Approval:	March 2022
Review date:	March 2025
Author(s):	• Joanne Collins- Specialist pharmacist- GI/Supportive care<br>• Dr Jorge Barriuso- Consultant Medical Oncologist<br>• Sarah Madden- HIPEC Service Manager for Peritoneal Tumour Service

This regimen is restricted to consultant colorectal oncologist use only.

Regimen Title

Mitomycin Intraoperative Intraperitoneal Chemotherapy in the treatment of Pseudomyxoma Peritonei (PMP), Metastatic Colon Cancer limited to the Peritoneal Cavity and Primary Appendix Adenocarcinoma.

Drug selection

There is a paucity of data to guide the selection of which intraperitoneal chemotherapy agent is used and the guidance below reflects established clinical practice at the Christie and consensus between prescribing oncologists.

It would be expected that the majority of patients treated will follow the guidance. However, it is accepted that individual patient circumstance may occasionally dictate a different treatment approach. If a deviation from guidelines is considered a discussion between prescribers would be best practice.

For Peer Review

Disease setting	Treatment line	Drug of choice
**PMP**	1st line	Mitomycin
	2nd line	Oxaliplatin
	3rd line	Mitomycin 20% dose reduction
**Colorectal peritoneal disease**	1st line	No prior systemic Oxaliplatin exposure – Oxaliplatin<br><br>or<br><br>Prior systemic Oxaliplatin exposure* – Mitomycin
	2nd line	Opposite regimen to that used first line e.g., Mitomycin if previous Oxaliplatin HIPEC
	3rd line	Mitomycin - 20% dose reduction
**Appendix adenoCa**	1st line	Mitomycin
	2nd line	Oxaliplatin
**Other e.g., Goblet cell carcinoid**	Management as per colorectal cancer	

*Based on increased risk of allergic reaction with prior exposure to Oxaliplatin

For Peer Review

Eligibility

- All cases considered for cytoreductive surgery (CRS) with hyperthermic intraperitoneal chemotherapy (HIPEC) must be reviewed in the specialist peritoneal tumour service MDT
- Mucinous or non-mucinous peritoneal carcinomatosis arising from an appendiceal or colorectal primary tumour)
- Adequate marrow reserve (ANC ≥1.5 x 10^9/L, platelets greater than 100 x 10^9/L)
- Adequate renal (creatinine less than or equal to 1.5 x ULN) and liver function (bilirubin ≤1.5 x ULN; AST/ Alkaline Phosphatase ≤5 x ULN)

Treatment Intent

Cytoreductive surgery and HIPEC is a radical treatment performed with the aim of achieving complete resection of all visible disease. It is a potentially curative treatment.

Contraindications

- ECOG > 2
- Non appendiceal or colorectal tumour
- Unresectable disease on preoperative imaging
- Extra-abdominal metastases
- Multifocal malignant small bowel obstruction
- Co-morbidities precluding extensive surgery (renal failure, cardiac disease, COPD, irreversible haematological disorders, and other)
- INR >1.4

Cautions

- Age > 70 years
- Extensive disease not amenable for R0/1 resection
- Synchronous liver metastases
- Disease progression while on chemotherapy
- High-grade adenocarcinoma
- Bilateral hydronephrosis

Expected toxicities

Systemic absorption of intraperitoneal mitomycin may lead to drug-induced toxicity: Most of the side effects are from having the operation rather than directly having the chemotherapy.

The surgery has serious complications reported in the international literature of around 30% (although the complication rates reported by the Christie compare very favourably to this):

- **Inflammation of the pancreas** (pancreatitis), about 6 to 7 patients in every 100 will develop this problem

For Peer Review

- **Postoperative bleeding or the development of a leak from the bowel** through tissue damage, about 4 to 5 patients in every 100 will be affected
- **Myelosuppression, including neutropenia**- therefore risk of infection or bleeding will affect 1 in 10 patients.

The side effects from the chemotherapy agents may include:

- **Nausea and vomiting**- antiemetics will be given if necessary.
- **Infection** – increased risk of intra-abdominal infection (peritonitis).
- **Delayed healing**
- **Lethargy/tiredness**
- **Diarrhoea**
- **Mucositis/stomatitis**

Late Toxicities:

Given the limited systemic exposure to mitomycin late organ specific toxicities are rare and would not be expected. When mitomycin is used systemically late toxicities can include:

- **Pulmonary toxicity**: Mitomycin is associated with pulmonary toxicity consisting of dyspnoea and non-productive cough, with an incidence of 3-12%. Threshold dose for pulmonary toxicity is 50-60mg/m^2.
- **Renal toxicity**: Mitomycin is associated with a syndrome of renal failure and **microangiopathic haemolytic anaemia**, with an incidence of 10%. Threshold dose for this syndrome is 50-60mg/m^2, usually appearing after 6 months of therapy.

A full list of expected toxicities can be viewed at the Electronic Medicines Compendium

Overview of treatment programme

The total mitomycin dose is 35mg/m^2 (capped at 70mg). If patient has had previous mitomycin exposure, consider 20% dose reduction to 28mg/m^2.

For Peer Review

Treatment is administered in surgical theatres:

Time	*Drug*	*Dose*	*Route*	*Administration*
**Cycle 1**				
T=0	Mitomycin	11.66mg/m^2	Intraperitoneal (syringe)	Administer as an intraperitoneal infusion with the carrier fluid. To flush solution out at time = 90 minutes.
T=30 mins	Mitomycin	11.66mg/m^2	Intraperitoneal (syringe)	
T=60 mins	Mitomycin	11.66mg/m^2	Intraperitoneal (syringe)	

Once only treatment

- Treatment to be provided in three equal dose syringes, which will be added to the peritoneal circulation at time = 0, 30 minutes and 60 minutes.
- Intraperitoneal mitomycin is mixed in 2 L/m² of 1.5% dextrose DIANEAL® PD4 peritoneal dialysis solution perfused for 90 minutes at intraperitoneal temperature >42°C using closed abdominal technique and hyperthermia pump, flow rate 1.2L/min.

For Peer Review

Additional medication

For most patients this regimen has low/moderate emetogenicity.

Checklist of initial investigations and work-up prior to start of treatment

Note that patient will be reviewed in pre-op clinic by anaesthetic team who will undertake a full medical history and physiological assessment. Further investigations may be requested at the discretion of the anaesthetist and oncology team.

☐ *Staging CT thorax/abdo/pelvis*

☐ *Medical history*

☐ *Physical assessment*

- *FBC, U&Es, LFTs, calculate creatinine clearance (CrCL)*
- *Tumour markers: CEA*
- *Ensure up to date height and weight are recorded*
- *Prescribe chemotherapy*

Dose modifications

HIPEC is a once only course of mitomycin, so cumulative toxic effects are not usually a concern. Any dose adjustments are at the discretion of the prescribing consultant oncologist.

**Haematological**			
Neutrophils		*Plt*	*Action*
≥1.5	***and***	*≥100*	*Go ahead with chemotherapy*
<1.5	**and/or**	<100	Consultant decision

**Renal impairment**	
Creatinine Clearance (ml/min)	*Action*
≥60	Full dose
<60	Consultant decision

**Hepatic impairment**
There is no specific guidance for mitomycin dose reductions in liver impairment exists, monitor carefully. Elevated AST levels may produce a prolonged plasma half-life.

Post treatment follow-up

- Patient will be followed up by the surgical team as an outpatient following their discharge from the hospital and adequate recovery time.
- Oncological follow up will be with the patient's local team.

For Peer Review

Change log

Date	Version no	Author	Changes
Feb 2019	1.0	Joanne Collins	-
March 2022	1.1	Joanne Collins	Regimen title updated.<br>MMC renal dosing changed for consistency with other MMC-containing protocols.<br>HIPEC technique used is a closed technique, rather than open.<br>References updated

For Peer Review

References

1. Chua TC, Moran BJ, Sugarbaker PH, et al. Early- and long-term outcome data of patients with pseudomyxoma peritonei from appendiceal origin treated by a strategy of cytoreductive surgery and hyperthermic intraperitoneal chemotherapy. J Clin Oncol 2012;30(20):2449–56.

2. Verwaal VJ, Bruin S, Boot H, van Slooten G, van Tinteren H. 8-year follow-up of randomized trial: cytoreduction and hyperthermic intraperitoneal chemotherapy versus systemic chemotherapy in patients with peritoneal carcinomatosis of colorectal cancer. Ann Surg Oncol 2008;15(9):2426–32.

3. Elias D, Gilly F, Boutitie F, et al. Peritoneal colorectal carcinomatosis treated with surgery and perioperative intraperitoneal chemotherapy: retrospective analysis of 523 patients from a multicentric French study. J Clin Oncol 2010;28(1):63–8.

4. Elias D, Lefevre JH, Chevalier J, et al. Complete cytoreductive surgery plus intraperitoneal chemohyperthermia with oxaliplatin for peritoneal carcinomatosis of colorectal origin. J Clin Oncol 2009;27(5):681–5.

5. The Christie NHS Foundation Trust. HIPEC - Guidance for the selection of IP chemotherapy agent

6. The Christie NHS Foundation Trust Peritoneal Tumour Service: HIPEC Protocol- mitomycin

7. The Christie NHS Foundation Trust Peritoneal Tumour Service: 102 Hyperthermic Intraperitoneal Chemotherapy (HIPEC) Information for patients (Sept 2014)

8. BC Cancer protocol. BCCA Protocol Summary for Hyperthermic Intraperitoneal Chemotherapy (HIPEC) for Patients with Peritoneal Carcinomatosis from Limited Advanced Colorectal and Appendiceal Carcinomas Using Oxaliplatin and Fluorouracil (5-FU) (revised Oct 2016). Available at:

For Peer Review

http://www.bccancer.bc.ca/chemotherapy-protocols-site/Documents/Gastrointestinal/GIHIPEC_Protocol.pdf

9. Kusamura S, Barretta F, Yonemura Y, Sugarbaker PH, Moran BJ, Levine EA, Goere D, Baratti D, Nizri E, Morris DL, Glehen O, Sardi A, Barrios P, Quénet F, Villeneuve L, Gómez-Portilla A, de Hingh I, Ceelen W, Pelz JOW, Piso P, González-Moreno S, Van Der Speeten K, Deraco M; Peritoneal Surface Oncology Group International (PSOGI) and the French National Registry of Rare Peritoneal Surface Malignancies (RENAPE). The Role of Hyperthermic Intraperitoneal Chemotherapy in Pseudomyxoma Peritonei After Cytoreductive Surgery. JAMA Surg. 2021 Mar 1;156(3): e206363.

For Peer Review

The Christie NHS
NHS Foundation Trust

Document Title:	***Treatment Protocol for Oxaliplatin Intraoperative Intraperitoneal Chemotherapy (HIPEC)***
Approved by:	Dr Mike Braun- Consultant medical oncologist<br>Peritoneal Service MDT
Version no:	1.1
Date of Approval:	March 2022
Review date:	March 2025
Author(s):	• Joanne Collins- Specialist pharmacist- GI/Supportive care<br>• Dr Jorge Barriuso- Consultant Medical Oncologist<br>• Sarah Madden- HIPEC Service Manager for Peritoneal Tumour Service

For Peer Review

This regimen is restricted to consultant colorectal oncologist use only.

Regimen Title

Oxaliplatin Intraoperative Intraperitoneal Chemotherapy in the treatment of Pseudomyxoma Peritonei (PMP), Metastatic Colon Cancer limited to the Peritoneal Cavity and Primary Adenocarcinoma of the Appendix.

Drug selection

There is a paucity of data to guide the selection of which intraperitoneal chemotherapy agent is used and the guidance below reflects established clinical practice at the Christie and consensus between prescribing oncologists.

It would be expected that the majority of patients treated will follow the guidance. However, it is accepted that individual patient circumstance may occasionally dictate a different treatment approach. If a deviation from guidelines is considered a discussion between prescribers would be best practice.

DPYD testing

Routine germline testing for mutations within the DPYD gene was recommended by Genomics England in 2020. Mutations in this gene, which metabolises 5FU and Capecitabine chemotherapy, can result in severe and potentially life-threatening side-effects. All patients being considered for chemotherapy using these agents are now required to have DPYD testing performed prior to treatment being administered. The only exclusion would be patients who have previously tolerated 5FU or Capecitabine chemotherapy without significant toxicity and are therefore not at high risk of severe toxicity.

Any patient planned to receive first-line 5FU/ Oxaliplatin HIPEC, based on the table below, should have been assessed for DPYD germline mutations. It should be noted that 5FU/ Oxaliplatin is currently considered an option for all patients either as 1st line or as re-challenge treatment.

At a patient's initial consultation with the surgical team, it should be established:

1. Whether a patient has already had germline DPYD testing performed at the referring hospital. If testing has been performed a copy of the report should be uploaded to CWP.
2. Whether a patient has previously received 5FU or Capecitabine chemotherapy and how they tolerated treatment. If a patient has received either of these drugs without developing severe toxicity, they do not have a significant DPYD germline mutation. Any patient who has experienced significant side-effects e.g., treatment dose reduced or discontinued, should have DPYD testing performed.

If patients have NOT been tested for DPYD germline mutations and have not received 5FU or capecitabine, a request for DPYD germline testing is required to inform the subsequent choice of HIPEC regimen. Requests can occur at the patient's referring hospital or at the Christie. Requests at the Christie should use the following form: - https://hive.xchristie.nhs.uk/Interact/Pages/Content/Document.aspx?id=14365&SearchId=

Choice of HIPEC treatment will vary dependent upon the factors described in the table below and the DPYD test result. Patients who are homozygous for DPYD

John Wiley & Sons, Inc.

mutations and are at risk of severe toxicity from 5FU chemotherapy should be considered for Mitomycin HIPEC. Patients who are heterozygous for a mutation could be considered for 5FU containing HIPEC dependent upon the clinical scenario and the specific advice provided in the DPYD mutation analysis report.

Disease setting	Treatment line	Drug of choice
PMP	1st line	Mitomycin
	2nd line	Oxaliplatin
	3rd line	Mitomycin 20% dose reduction
Colorectal peritoneal disease	1st line	No prior systemic Oxaliplatin exposure – Oxaliplatin<br>or<br>Prior systemic Oxaliplatin exposure* – Mitomycin
	2nd line	Opposite regimen to that used first line e.g., Mitomycin if previous Oxaliplatin HIPEC
	3rd line	Mitomycin - 20% dose reduction
Appendix adenoCa	1st line	Mitomycin
	2nd line	Oxaliplatin
Other e.g., Goblet cell carcinoid	Management as per colorectal cancer	

*Based on increased risk of allergic reaction with prior exposure to Oxaliplatin

For Peer Review

Eligibility

- All cases considered for cytoreductive surgery (CRS) with hyperthermic intraperitoneal chemotherapy (HIPEC) must be reviewed in the specialist peritoneal tumour service MDT
- Mucinous or non-mucinous peritoneal carcinomatosis arising from an appendiceal or colorectal primary tumour
- Adequate marrow reserve (ANC ≥1.5 x 10^9/L, platelets greater than 100 x 10^9/L)
- Adequate renal (creatinine less than or equal to 1.5 x ULN) and liver function (bilirubin ≤1.5 x ULN; AST/ Alkaline Phosphatase ≤5 x ULN)

John Wiley & Sons, Inc.

Treatment Intent

Cytoreductive surgery and HIPEC is a radical treatment performed with the aim of achieving complete resection of all visible disease. It is a potentially curative treatment.

Contraindications

- ECOG > 2
- Allergic reaction or significant documented toxicity from pre-existing platinum-based therapies
- Non appendiceal or colorectal tumour
- Unresectable disease on preoperative imaging
- Extra-abdominal metastases
- Multifocal malignant small bowel obstruction
- Co-morbidities precluding extensive surgery (renal failure, cardiac disease, COPD, irreversible haematological disorders, and other)
- INR >1.4

Cautions

- Age > 70 years
- Extensive disease not amenable for R0/1 resection
- Synchronous liver metastases
- Disease progression while on chemotherapy
- High-grade adenocarcinoma
- Bilateral hydronephrosis

Avoid use in patients with known DPD deficiency.
Severe diarrhoea and/or severe mucositis early in the first treatment cycle can be the first presenting toxicity due to DPD enzyme deficiency, in which case potentially fatal neutropenia can quickly follow.

Expected toxicities

Most of the side effects are from having the operation rather than directly having the chemotherapy.

The surgery has serious complications reported in the international literature of around 30% (although the complication rates reported by the Christie compare very favourably to this):

For Peer Review

- **Inflammation of the pancreas** (pancreatitis), about 6 to 7 patients in every 100 will develop this problem
- **Postoperative bleeding or the development of a leak from the bowel** through tissue damage, about 4 to 5 patients in every 100 will be affected
- **Myelosuppression, including neutropenia**- therefore risk of infection or bleeding will affect 1 in 10 patients.

The side effects from the chemotherapy agents may include:

- **Nausea and vomiting**- antiemetic medication will be given if necessary.
- **Infection –** Increased risk of intra-abdominal infection (peritonitis).
- **Delayed healing**
- **Lethargy/tiredness**
- **Diarrhoea**
- **Mucositis/stomatitis**
- **Allergic reactions (oxaliplatin)**
- **Neuropathy (oxaliplatin)**
- **Coronary artery spasm (fluorouracil)**
- **Severe 5-FU toxicity due to DPD deficiency (see above)**

A full list of expected toxicities can be viewed at the Electronic Medicines Compendium

For Peer Review

Overview of treatment programme

Consider dose capping chemotherapy at BSA 2.2m^2.

Treatment is administered in surgical theatres:

Time	*Drug*	*Dose*	*Route*	*Diluent*	*Administration*
**Day 1**					
T=0	Calcium folinate	50mg (Flat dose)	**IV bolus**	-	To be administered 60 minutes *before* intraperitoneal chemotherapy.
T=0 mins	Fluorouracil*	400mg/m^2	**IV infusion** (over 60 minutes)	250mL sodium chloride 0.9%	
T=60 mins	Oxaliplatin	368mg/m^2	**Intraperitoneal** (Perfused for 30 minutes)	-	For heated **INTRAPERITONEAL** perfusion with the carrier fluid. Dose to be prepared in an empty 5% Glucose bag.
**Once only treatment**					

- *The fluorouracil infusion is flushed through with 50mL sodium chloride 0.9%.
- Intraperitoneal oxaliplatin is mixed in 2 L/m² of 1.5% dextrose DIANEAL® PD4 peritoneal dialysis solution perfused for 30 minutes at intraperitoneal temperature >42°C using closed abdomen technique and hyperthermia pump, flow rate 1.2L/min.

Extravasation:

Fluorouracil is an inflammatant

Refer to the local Extravasation Guidelines

Additional medication

For most patients this regimen has low/moderate emetogenicity.

John Wiley & Sons, Inc.

For Peer Review

Checklist of initial investigations and work-up prior to start of treatment

Note that patients will be reviewed in pre-op clinic by anaesthetic team who will undertake a full medical history and physiological assessment. Further investigations may be requested at the discretion of the anaesthetist and oncology team.

- ☐ *Staging CT thorax/abdo/pelvis*
- ☐ *Medical history*
- ☐ *Physical assessment*
- ☐ *FBC, U&Es, LFTs, calculate creatinine clearance (CrCL)*
- ☐ *Tumour markers: CEA*
- ☐ *Ensure up to date height and weight are recorded*
- ☐ *Prescribe chemotherapy*

For Peer Review

Dose modifications

HIPEC is a once only course, so cumulative adverse effects are not usually a concern. Any dose adjustments are at the discretion of the prescribing consultant oncologist.

**Haematological**			
Neutrophils		*Plt*	*Action*
≥1.5	***and***	*≥100*	*Go ahead with chemotherapy*
<1.5	**and/or**	<100	Consultant decision

**Renal impairment**	
Creatinine Clearance (ml/min)	*Action*
≥50	Full dose
<50	Consultant decision

**Hepatic impairment**
Consultant decision.

John Wiley & Sons, Inc.

Post treatment follow-up

- Patient will be followed up by the surgical team as an outpatient following their discharge from the hospital and adequate recovery time.
- Oncological follow up will be with the patient's local team.

Change log

Date	Version no	Author	Changes
Feb 2019	1.0	Joanne Collins	-
March 2022	1.1	Joanne Collins	Regimen title updated.<br><br>DPYD testing information included.<br><br>HIPEC technique used is a closed technique, rather than open.<br><br>Renal dosing- Consultant decision where CrCl is <50ml/min.<br><br>References updated.

For Peer Review

References

1. Chua TC, Moran BJ, Sugarbaker PH, et al. Early- and long-term outcome data of patients with pseudomyxoma peritonei from appendiceal origin treated by a strategy of cytoreductive surgery and hyperthermic intraperitoneal chemotherapy. J Clin Oncol 2012;30(20):2449–56.

2. Verwaal VJ, Bruin S, Boot H, van Slooten G, van Tinteren H. 8-year follow-up of randomized trial: cytoreduction and hyperthermic intraperitoneal chemotherapy versus systemic chemotherapy in patients with peritoneal carcinomatosis of colorectal cancer. Ann Surg Oncol 2008;15(9):2426–32.

3. Elias D, Gilly F, Boutitie F, et al. Peritoneal colorectal carcinomatosis treated with surgery and perioperative intraperitoneal chemotherapy: retrospective analysis of 523 patients from a multicentric French study. J Clin Oncol 2010;28(1):63–8.

4. Elias D, Lefevre JH, Chevalier J, et al. Complete cytoreductive surgery plus intraperitoneal chemohyperthermia with oxaliplatin for peritoneal carcinomatosis of colorectal origin. J Clin Oncol 2009;27(5):681–5.

5. The Christie NHS Foundation Trust. HIPEC - Guidance for the selection of IP chemotherapy agent

6. The Christie NHS Foundation Trust Peritoneal Tumour Service: HIPEC Protocol- oxaliplatin

7. The Christie NHS Foundation Trust Peritoneal Tumour Service: 102 Hyperthermic Intraperitoneal Chemotherapy (HIPEC) Information for patients (Sept 2014)

8. BC Cancer protocol. BCCA Protocol Summary for Hyperthermic Intraperitoneal Chemotherapy (HIPEC) for Patients with Peritoneal Carcinomatosis from Limited Advanced Colorectal and Appendiceal Carcinomas Using Oxaliplatin and Fluorouracil (5-FU) (revised Oct 2016). Available at: http://www.bccancer.bc.ca/chemotherapy-protocols-site/Documents/Gastrointestinal/GIHIPEC_Protocol.pdf

For Peer Review

9. Kusamura S, Barretta F, Yonemura Y, Sugarbaker PH, Moran BJ, Levine EA, Goere D, Baratti D, Nizri E, Morris DL, Glehen O, Sardi A, Barrios P, Quénet F, Villeneuve L, Gómez-Portilla A, de Hingh I, Ceelen W, Pelz JOW, Piso P, González-Moreno S, Van Der Speeten K, Deraco M; Peritoneal Surface Oncology Group International (PSOGI) and the French National Registry of Rare Peritoneal Surface Malignancies (RENAPE). The Role of Hyperthermic Intraperitoneal Chemotherapy in Pseudomyxoma Peritonei After Cytoreductive Surgery. JAMA Surg. 2021 Mar 1;156(3): e206363.

For Peer Review

John Wiley & Sons, Inc.

MANCHESTER 1824
The University of Manchester

The Christie NHS
NHS Foundation Trust

Colorectal and Peritoneal Oncology Centre

Department 1, 4, 6, 10
The Christie Hospital NHS Foundation Trust
550 Wilmslow Road, Manchester, M20 4BX

27th May 2024

Answers provided to reviewer comments on invited manuscript PSOG-2024-0802

"**Pre-operative chemotherapy response and survival in patients with colorectal cancer peritoneal metastases**

JSO Special Issue on Peritoneal Surface Malignancy.

Reviewer Comments to the Author:

Reviewer: 1

1. Authors state in the Introduction, 3rd paragraph that systemic chemotherapy response before CRS and its impact on survival has only recently been studied in 30 patients (reference 14) by Sousa et al. Other manuscripts have, by retrospective analysis, shown this phenomenon clearly. The manuscript by Bijelic et al. (Bijelic L, Kumar AS, Stuart OA, Sugarbaker PH. Systemic chemotherapy prior to cytoreductive surgery and HIPEC for carcinomatosis from appendix cancer. Impact on perioperative outcomes and short-term survival. Gastroenterol Res Pract Volume 2012; 2012. Article ID 163284) may be the first study to show improved survival with CRS and HIPEC when there was response or near-complete response to preoperative systemic chemotherapy. Also, these references -- (Passot G, You B, Boschetti G, et al. Pathologic response to neoadjuvant chemotherapy: a new prognosis tool for the curative management of peritoneal colorectal carcinomatosis. Ann Surg Oncol 2014;21:2608-2614), (Sugarbaker PH, Chang D. Revised prognostic indicators for treatment of lymph node positive colorectal peritoneal metastases. J Surg Oncol 2022;125(5):889-9000), and (Ghabra S, Desale S, Sugarbaker PH. Clinical and histopathologic features of 35 patients treated for colorectal peritoneal metastases who survived 5 years. Dis Colon Rectum 2023;66(10):1329-1338) provide information regarding the impact of a response to chemotherapy prior to CRS and HIPEC.

We thank you for this comment and agree to address this in our manuscript. We have expanded our literature search and included the references provided, however made a distinction between studies that include colorectal cancer peritoneal metastases (CRPM) versus those that refer to

For Peer Review

peritoneal metastases from other tumour origin (e.g.: appendix). We feel this is an important distinction given the spectrum of appendiceal malignancy versus CRPM.

2. In Materials and methods, pages 8 and 9 - The authors describe chemotherapy response on CT imaging and also chemotherapy response by tumour regression grade of the postoperative histology. I don't see in their data where the changes in the imaging were used to generate data regarding the impact of preoperative chemotherapy on survival. If radiologic parameters were used, the radiologic criteria for response should be defined within the Materials and methods. Precise definition of lesions seen on CT, PET-CT or MRI is not straightforward and requires a dedicated radiologist. Accurate assessment of response of peritoneal metastases is even more complex and less well defined. My suggestion to the authors is to delete or somehow modify the description of the use of CT-TAP as an assessment of tumour response. Maybe a part of their overall clinical assessment but not used in the results to generate data.

We thank you for this valid comment, which we have considered very carefully. All CRPM patients who receive pre-operative chemotherapy before CRS have imaging (CT TAP +/- PET scan) before the start of chemotherapy and after completion of chemotherapy. All imaging is discussed at the Peritoneal Tumour MDT with a group of dedicated specialist peritoneal tumour radiologists, peritoneal surgeons, medical and clinical oncologists, with more than 20 years experience in peritoneal malignancies. Even though precise definition of response can be difficult (e.g. for the purpose of clinical trials), we feel that the methods used in this clinical setting (review peritoneal tumour bulk, radiologic assessment of PCI and assessment of visible marker lesions) is a good method to assess response in patients were this is clearly evident. In the present study, patients where chemotherapy response is not clearly evident (e.g. stable or progressive disease) are not included in the chemotherapy response category. In addition, where histology reporting of tumour response correlated, these patients were included in the chemotherapy response category. We feel this is an important part of our assessment and highlights how other intuitions could adopt a similar and effective system. We have previously published peritoneal radiological recurrence patterns following CRS + HIPEC with CT assessment (1).

3. The authors include with the manuscript 2 protocols. One for investigation of mitomycin C for HIPEC. A second protocol for investigation of HIPEC-oxaliplatin is included with the manuscript. These were to be placed as supplemental files. I suggest that these protocols be published in a journal that is specifically designed for the presentation of clinical trials. In my opinion, these 2 protocols are not appropriate for publication in the JSO PSOGI-Venice-2023 Special Issue.

However, I have no experience regarding the publication of protocols as part of a manuscript. Perhaps it is highly desirable. This is an issue that needs clarification between the JSO administration and the authors for a definitive decision.

We thank you for this comment. We feel that this information may have been useful for reviewer comments to acknowledge our practice, but agree this is not required for the final publication.

Minor comments:

1. Abstract, page 4, line 1 - Replace the word "informed" with "provided". *Changed*
2. Implications for practice, page 5, line 6 - Fix sentence to read, "We also assessed adverse......" *Changed*

We hope that our responses are considered appropriate and we hope to hear from you soon.

Yours sincerely,

Dr Nadina Tinsley

Clinical Research Fellow

Colorectal Peritoneal Oncology Centre at the Christie NHS Foundation Trust

Division of Cancer Sciences, University of Manchester

Telephone 07743565274

Email nadina.tinsley1@nhs.net

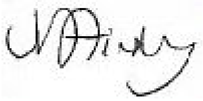

References

1. *Hassan S, Malcomson L, Soh YJ, Wilson MS, Clouston H, O'Dwyer ST, et al. Patterns and Timing of Recurrence following CRS and HIPEC in Colorectal Cancer Peritoneal Metastasis. Eur J Surg Oncol [Internet]. 2023 Jan;49(1):202–8. Available from: https://linkinghub.elsevier.com/retrieve/pii/S0748798322005613*

For Peer Review
