# Supplementary figures and images for: Preoperative chemotherapy response and survival in patients with colorectal cancer peritoneal metastases
Source: J Surg Oncol. 2024 Jul 16;130(6):1422–32. doi: 10.1002/jso.27776 (PMC11826003; doi:10.1002/jso.27776)

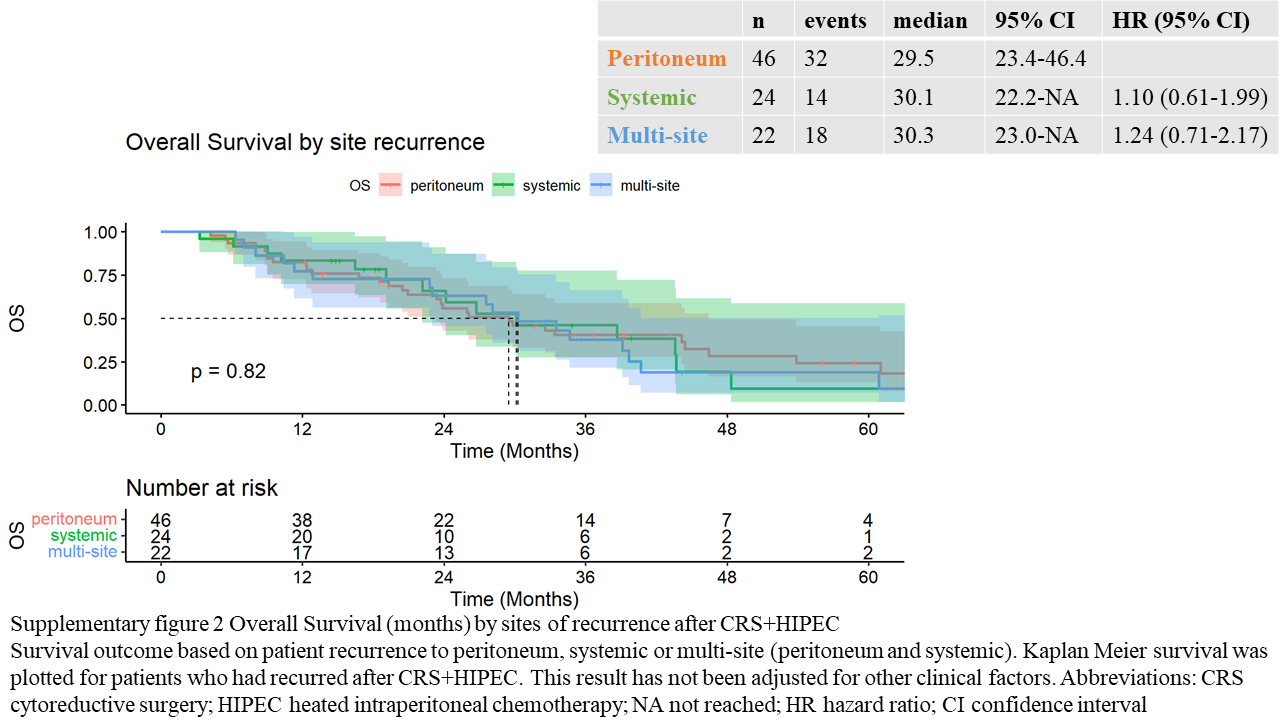

Supplement: Supplementary file 2 — Supporting information. [file JSO-130-1422-s006.TIF]

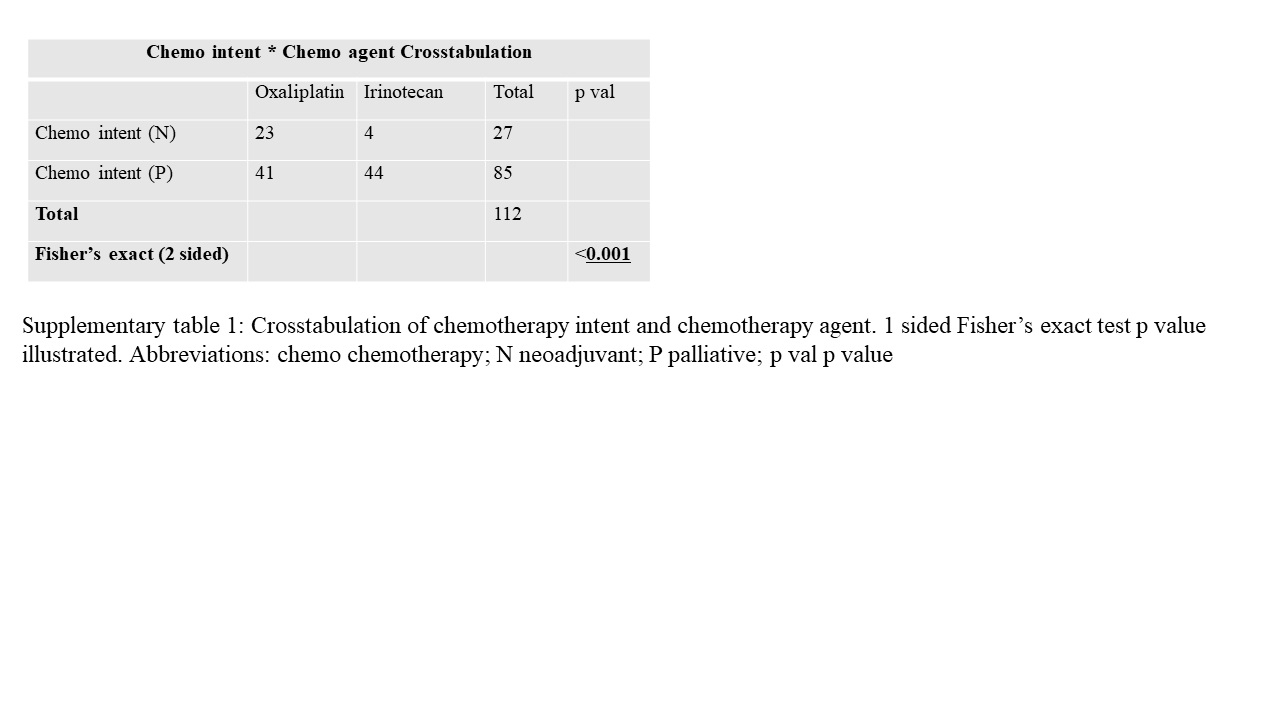

Supplement: Supplementary file 3 — Supporting information. [file JSO-130-1422-s005.TIF]

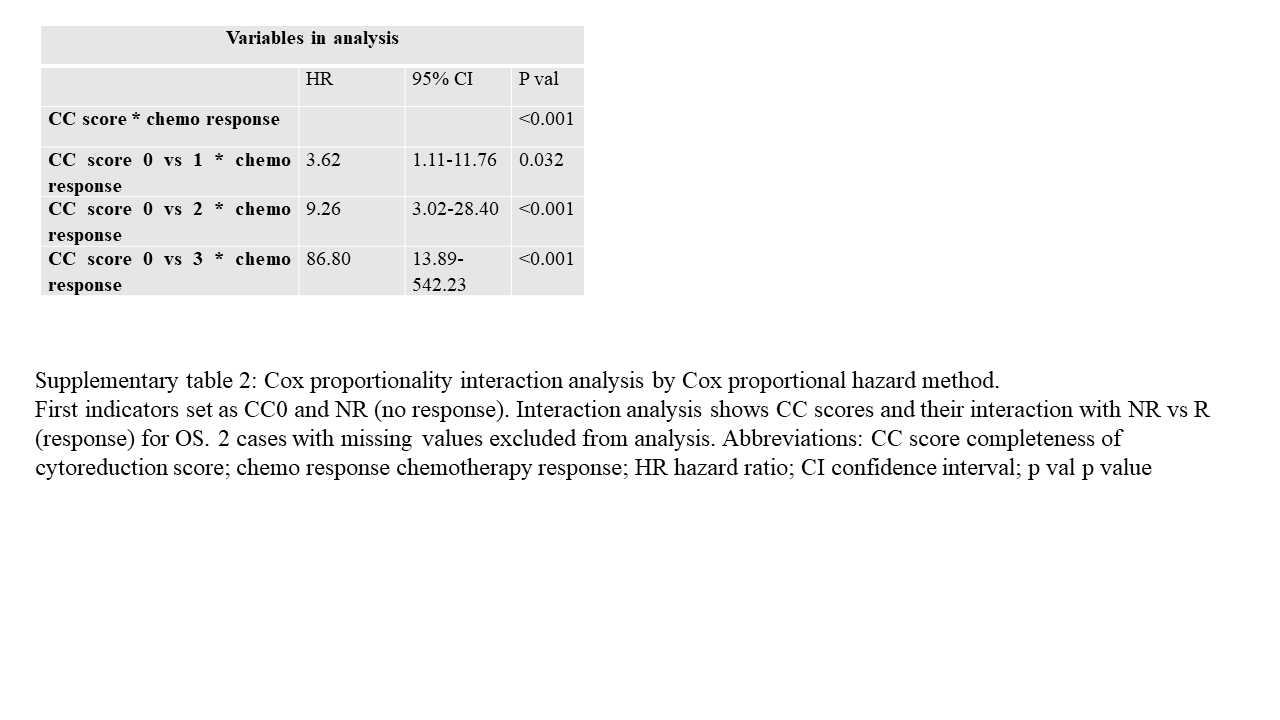

Supplement: Supplementary file 4 — Supporting information. [file JSO-130-1422-s002.TIF]
