# Supplementary material for: Preoperative chemotherapy response and survival in patients with colorectal cancer peritoneal metastases
Source: J Surg Oncol. 2024 Jul 16;130(6):1422–32. doi: 10.1002/jso.27776 (PMC11826003; doi:10.1002/jso.27776)
Supplement: Supplementary file 5 — Appendix 1: Local HIPEC protocols. [file JSO-130-1422-s001.pdf]

The Christie NHS
NHS Foundation Trust

Document Title:	***Treatment Protocol for Mitomycin Intraoperative Intraperitoneal Chemotherapy (HIPEC)***
Approved by:	Dr Mike Braun- Consultant medical oncologist<br>Peritoneal Service MDT
Version no:	1.1
Date of Approval:	March 2022
Review date:	March 2025
Author(s):	• Joanne Collins- Specialist pharmacist- GI/Supportive care<br>• Dr Jorge Barriuso- Consultant Medical Oncologist<br>• Sarah Madden- HIPEC Service Manager for Peritoneal Tumour Service

This regimen is restricted to consultant colorectal oncologist use only.

Regimen Title

Mitomycin Intraoperative Intraperitoneal Chemotherapy in the treatment of Pseudomyxoma Peritonei (PMP), Metastatic Colon Cancer limited to the Peritoneal Cavity and Primary Appendix Adenocarcinoma.

Drug selection

There is a paucity of data to guide the selection of which intraperitoneal chemotherapy agent is used and the guidance below reflects established clinical practice at the Christie and consensus between prescribing oncologists.

It would be expected that the majority of patients treated will follow the guidance. However, it is accepted that individual patient circumstance may occasionally dictate a different treatment approach. If a deviation from guidelines is considered a discussion between prescribers would be best practice.

Disease setting	Treatment line	Drug of choice
**PMP**	1st line	Mitomycin
	2nd line	Oxaliplatin
	3rd line	Mitomycin 20% dose reduction
**Colorectal peritoneal disease**	1st line	No prior systemic Oxaliplatin exposure – Oxaliplatin<br><br>or<br><br>Prior systemic Oxaliplatin exposure* – Mitomycin
	2nd line	Opposite regimen to that used first line e.g., Mitomycin if previous Oxaliplatin HIPEC
	3rd line	Mitomycin - 20% dose reduction
**Appendix adenoCa**	1st line	Mitomycin
	2nd line	Oxaliplatin
**Other e.g., Goblet cell carcinoid**	Management as per colorectal cancer	

*Based on increased risk of allergic reaction with prior exposure to Oxaliplatin

Eligibility

- All cases considered for cytoreductive surgery (CRS) with hyperthermic intraperitoneal chemotherapy (HIPEC) must be reviewed in the specialist peritoneal tumour service MDT
- Mucinous or non-mucinous peritoneal carcinomatosis arising from an appendiceal or colorectal primary tumour)
- Adequate marrow reserve (ANC ≥1.5 x 10^9/L, platelets greater than 100 x 10^9/L)
- Adequate renal (creatinine less than or equal to 1.5 x ULN) and liver function (bilirubin ≤1.5 x ULN; AST/ Alkaline Phosphatase ≤5 x ULN)

Treatment Intent

Cytoreductive surgery and HIPEC is a radical treatment performed with the aim of achieving complete resection of all visible disease. It is a potentially curative treatment**.**

Contraindications

- ECOG > 2
- Non appendiceal or colorectal tumour
- Unresectable disease on preoperative imaging
- Extra-abdominal metastases
- Multifocal malignant small bowel obstruction
- Co-morbidities precluding extensive surgery (renal failure, cardiac disease, COPD, irreversible haematological disorders, and other)
- INR >1.4

Cautions

- Age > 70 years
- Extensive disease not amenable for R0/1 resection
- Synchronous liver metastases
- Disease progression while on chemotherapy
- High-grade adenocarcinoma
- Bilateral hydronephrosis

Expected toxicities

Systemic absorption of intraperitoneal mitomycin may lead to drug-induced toxicity: Most of the side effects are from having the operation rather than directly having the chemotherapy.

The surgery has serious complications reported in the international literature of around 30% (although the complication rates reported by the Christie compare very favourably to this):

- **Inflammation of the pancreas** (pancreatitis), about 6 to 7 patients in every 100 will develop this problem

- **Postoperative bleeding or the development of a leak from the bowel** through tissue damage, about 4 to 5 patients in every 100 will be affected
- **Myelosuppression, including neutropenia**- therefore risk of infection or bleeding will affect 1 in 10 patients.

The side effects from the chemotherapy agents may include:

- **Nausea and vomiting**- antiemetics will be given if necessary.
- **Infection** – increased risk of intra-abdominal infection (peritonitis).
- **Delayed healing**
- **Lethargy/tiredness**
- **Diarrhoea**
- **Mucositis/stomatitis**

Late Toxicities:

Given the limited systemic exposure to mitomycin late organ specific toxicities are rare and would not be expected. When mitomycin is used systemically late toxicities can include:

- **Pulmonary toxicity**: Mitomycin is associated with pulmonary toxicity consisting of dyspnoea and non-productive cough, with an incidence of 3-12%. Threshold dose for pulmonary toxicity is 50-60mg/m^2.
- **Renal toxicity**: Mitomycin is associated with a syndrome of renal failure and **microangiopathic haemolytic anaemia**, with an incidence of 10%. Threshold dose for this syndrome is 50-60mg/m^2, usually appearing after 6 months of therapy.

A full list of expected toxicities can be viewed at the Electronic Medicines Compendium

Overview of treatment programme

The total mitomycin dose is 35mg/m^2 (capped at 70mg). If patient has had previous mitomycin exposure, consider 20% dose reduction to 28mg/m^2.

Treatment is administered in surgical theatres:

Time	*Drug*	*Dose*	*Route*	*Administration*
**Cycle 1**				
T=0	Mitomycin	11.66mg/m^2	Intraperitoneal<br><br>(syringe)	Administer as an intraperitoneal infusion with the carrier fluid.
T=30 mins	Mitomycin	11.66mg/m^2	Intraperitoneal<br><br>(syringe)	To flush solution out at time = 90 minutes.
T=60 mins	Mitomycin	11.66mg/m^2	Intraperitoneal<br><br>(syringe)	
**Once only treatment**<br>• Treatment to be provided in three equal dose syringes, which will be added to the peritoneal circulation at time = 0, 30 minutes and 60 minutes.<br>• Intraperitoneal mitomycin is mixed in 2 L/m² of 1.5% dextrose DIANEAL® PD4 peritoneal dialysis solution perfused for 90 minutes at intraperitoneal temperature >42°C using closed abdominal technique and hyperthermia pump, flow rate 1.2L/min.				

Additional medication

For most patients this regimen has low/moderate emetogenicity.

Checklist of initial investigations and work-up prior to start of treatment

Note that patient will be reviewed in pre-op clinic by anaesthetic team who will undertake a full medical history and physiological assessment. Further investigations may be requested at the discretion of the anaesthetist and oncology team.

☐ *Staging CT thorax/abdo/pelvis*

☐ *Medical history*

☐ *Physical assessment*

- *FBC, U&Es, LFTs, calculate creatinine clearance (CrCL)*
- *Tumour markers: CEA*
- *Ensure up to date height and weight are recorded*
- *Prescribe chemotherapy*

Dose modifications

HIPEC is a once only course of mitomycin, so cumulative toxic effects are not usually a concern. Any dose adjustments are at the discretion of the prescribing consultant oncologist.

**Haematological**			
Neutrophils		*Plt*	*Action*
≥1.5	***and***	*≥100*	*Go ahead with chemotherapy*
<1.5	**and/or**	<100	Consultant decision

**Renal impairment**	
Creatinine Clearance (ml/min)	*Action*
≥60	Full dose
<60	Consultant decision

**Hepatic impairment**
There is no specific guidance for mitomycin dose reductions in liver impairment exists, monitor carefully. Elevated AST levels may produce a prolonged plasma half-life.

Post treatment follow-up

- Patient will be followed up by the surgical team as an outpatient following their discharge from the hospital and adequate recovery time.
- Oncological follow up will be with the patient's local team.

Change log

Date	Version no	Author	Changes
Feb 2019	1.0	Joanne Collins	-
March 2022	1.1	Joanne Collins	Regimen title updated.<br><br>MMC renal dosing changed for consistency with other MMC-containing protocols.<br><br>HIPEC technique used is a closed technique, rather than open.<br><br>References updated

References

1. Chua TC, Moran BJ, Sugarbaker PH, et al. Early- and long-term outcome data of patients with pseudomyxoma peritonei from appendiceal origin treated by a strategy of cytoreductive surgery and hyperthermic intraperitoneal chemotherapy. J Clin Oncol 2012;30(20):2449–56.

2. Verwaal VJ, Bruin S, Boot H, van Slooten G, van Tinteren H. 8-year follow-up of randomized trial: cytoreduction and hyperthermic intraperitoneal chemotherapy versus systemic chemotherapy in patients with peritoneal carcinomatosis of colorectal cancer. Ann Surg Oncol 2008;15(9):2426–32.

3. Elias D, Gilly F, Boutitie F, et al. Peritoneal colorectal carcinomatosis treated with surgery and perioperative intraperitoneal chemotherapy: retrospective analysis of 523 patients from a multicentric French study. J Clin Oncol 2010;28(1):63–8.

4. Elias D, Lefevre JH, Chevalier J, et al. Complete cytoreductive surgery plus intraperitoneal chemohyperthermia with oxaliplatin for peritoneal carcinomatosis of colorectal origin. J Clin Oncol 2009;27(5):681–5.

5. The Christie NHS Foundation Trust. HIPEC - Guidance for the selection of IP chemotherapy agent

6. The Christie NHS Foundation Trust Peritoneal Tumour Service: HIPEC Protocol- mitomycin

7. The Christie NHS Foundation Trust Peritoneal Tumour Service: 102 Hyperthermic Intraperitoneal Chemotherapy (HIPEC) Information for patients (Sept 2014)

8. BC Cancer protocol. BCCA Protocol Summary for Hyperthermic Intraperitoneal Chemotherapy (HIPEC) for Patients with Peritoneal Carcinomatosis from Limited Advanced Colorectal and Appendiceal Carcinomas Using Oxaliplatin and Fluorouracil (5-FU) (revised Oct 2016). Available at:

http://www.bccancer.bc.ca/chemotherapy-protocols-site/Documents/Gastrointestinal/GIHIPEC_Protocol.pdf

9. Kusamura S, Barretta F, Yonemura Y, Sugarbaker PH, Moran BJ, Levine EA, Goere D, Baratti D, Nizri E, Morris DL, Glehen O, Sardi A, Barrios P, Quénet F, Villeneuve L, Gómez-Portilla A, de Hingh I, Ceelen W, Pelz JOW, Piso P, González-Moreno S, Van Der Speeten K, Deraco M; Peritoneal Surface Oncology Group International (PSOGI) and the French National Registry of Rare Peritoneal Surface Malignancies (RENAPE). The Role of Hyperthermic Intraperitoneal Chemotherapy in Pseudomyxoma Peritonei After Cytoreductive Surgery. JAMA Surg. 2021 Mar 1;156(3): e206363.
