# Supplementary material for: Preoperative chemotherapy response and survival in patients with colorectal cancer peritoneal metastases
Source: J Surg Oncol. 2024 Jul 16;130(6):1422–32. doi: 10.1002/jso.27776 (PMC11826003; doi:10.1002/jso.27776)
Supplement: Supplementary file 6 — Supporting information. [file JSO-130-1422-s004.pdf]

The Christie NHS
NHS Foundation Trust

Document Title:	***Treatment Protocol for Oxaliplatin Intraoperative Intraperitoneal Chemotherapy (HIPEC)***
Approved by:	Dr Mike Braun- Consultant medical oncologist<br>Peritoneal Service MDT
Version no:	1.1
Date of Approval:	March 2022
Review date:	March 2025
Author(s):	• Joanne Collins- Specialist pharmacist- GI/Supportive care<br>• Dr Jorge Barriuso- Consultant Medical Oncologist<br>• Sarah Madden- HIPEC Service Manager for Peritoneal Tumour Service

This regimen is restricted to consultant colorectal oncologist use only.

Regimen Title

Oxaliplatin Intraoperative Intraperitoneal Chemotherapy in the treatment of Pseudomyxoma Peritonei (PMP), Metastatic Colon Cancer limited to the Peritoneal Cavity and Primary Adenocarcinoma of the Appendix.

Drug selection

There is a paucity of data to guide the selection of which intraperitoneal chemotherapy agent is used and the guidance below reflects established clinical practice at the Christie and consensus between prescribing oncologists.

It would be expected that the majority of patients treated will follow the guidance. However, it is accepted that individual patient circumstance may occasionally dictate a different treatment approach. If a deviation from guidelines is considered a discussion between prescribers would be best practice.

DPYD testing

Routine germline testing for mutations within the DPYD gene was recommended by Genomics England in 2020. Mutations in this gene, which metabolises 5FU and Capecitabine chemotherapy, can result in severe and potentially life-threatening side-effects. All patients being considered for chemotherapy using these agents are now required to have DPYD testing performed prior to treatment being administered. The only exclusion would be patients who have previously tolerated 5FU or Capecitabine chemotherapy without significant toxicity and are therefore not at high risk of severe toxicity.

Any patient planned to receive first-line 5FU/ Oxaliplatin HIPEC, based on the table below, should have been assessed for DPYD germline mutations. It should be noted that 5FU/ Oxaliplatin is currently considered an option for all patients either as 1st line or as re-challenge treatment.

At a patient's initial consultation with the surgical team, it should be established:

1. Whether a patient has already had germline DPYD testing performed at the referring hospital. If testing has been performed a copy of the report should be uploaded to CWP.
2. Whether a patient has previously received 5FU or Capecitabine chemotherapy and how they tolerated treatment. If a patient has received either of these drugs without developing severe toxicity, they do not have a significant DPYD germline mutation. Any patient who has experienced significant side-effects e.g., treatment dose reduced or discontinued, should have DPYD testing performed.

If patients have NOT been tested for DPYD germline mutations and have not received 5FU or capecitabine, a request for DPYD germline testing is required to inform the subsequent choice of HIPEC regimen. Requests can occur at the patient's referring hospital or at the Christie. Requests at the Christie should use the following form: - https://hive.xchristie.nhs.uk/Interact/Pages/Content/Document.aspx?id=14365&SearchId=

Choice of HIPEC treatment will vary dependent upon the factors described in the table below and the DPYD test result. Patients who are homozygous for DPYD

mutations and are at risk of severe toxicity from 5FU chemotherapy should be considered for Mitomycin HIPEC. Patients who are heterozygous for a mutation could be considered for 5FU containing HIPEC dependent upon the clinical scenario and the specific advice provided in the DPYD mutation analysis report.

**Disease setting**	**Treatment line**	**Drug of choice**
PMP	1st line	Mitomycin
	2nd line	Oxaliplatin
	3rd line	Mitomycin 20% dose reduction
Colorectal peritoneal disease	1st line	No prior systemic Oxaliplatin exposure – Oxaliplatin<br>or<br>Prior systemic Oxaliplatin exposure* – Mitomycin
	2nd line	Opposite regimen to that used first line e.g., Mitomycin if previous Oxaliplatin HIPEC
	3rd line	Mitomycin - 20% dose reduction
Appendix adenoCa	1st line	Mitomycin
	2nd line	Oxaliplatin
Other e.g., Goblet cell carcinoid	Management as per colorectal cancer	

*Based on increased risk of allergic reaction with prior exposure to Oxaliplatin

Eligibility

- All cases considered for cytoreductive surgery (CRS) with hyperthermic intraperitoneal chemotherapy (HIPEC) must be reviewed in the specialist peritoneal tumour service MDT
- Mucinous or non-mucinous peritoneal carcinomatosis arising from an appendiceal or colorectal primary tumour
- Adequate marrow reserve (ANC ≥1.5 x 10^9/L, platelets greater than 100 x 10^9/L)
- Adequate renal (creatinine less than or equal to 1.5 x ULN) and liver function (bilirubin ≤1.5 x ULN; AST/ Alkaline Phosphatase ≤5 x ULN)

Treatment Intent

Cytoreductive surgery and HIPEC is a radical treatment performed with the aim of achieving complete resection of all visible disease. It is a potentially curative treatment.

Contraindications

- ECOG > 2
- Allergic reaction or significant documented toxicity from pre-existing platinum-based therapies
- Non appendiceal or colorectal tumour
- Unresectable disease on preoperative imaging
- Extra-abdominal metastases
- Multifocal malignant small bowel obstruction
- Co-morbidities precluding extensive surgery (renal failure, cardiac disease, COPD, irreversible haematological disorders, and other)
- INR >1.4

Cautions

- Age > 70 years
- Extensive disease not amenable for R0/1 resection
- Synchronous liver metastases
- Disease progression while on chemotherapy
- High-grade adenocarcinoma
- Bilateral hydronephrosis

Avoid use in patients with known DPD deficiency.
Severe diarrhoea and/or severe mucositis early in the first treatment cycle can be the first presenting toxicity due to DPD enzyme deficiency, in which case potentially fatal neutropenia can quickly follow.

Expected toxicities

Most of the side effects are from having the operation rather than directly having the chemotherapy.

The surgery has serious complications reported in the international literature of around 30% (although the complication rates reported by the Christie compare very favourably to this):

- **Inflammation of the pancreas** (pancreatitis), about 6 to 7 patients in every 100 will develop this problem
- **Postoperative bleeding or the development of a leak from the bowel** through tissue damage, about 4 to 5 patients in every 100 will be affected
- **Myelosuppression, including neutropenia**- therefore risk of infection or bleeding will affect 1 in 10 patients.

The side effects from the chemotherapy agents may include:

- **Nausea and vomiting**- antiemetic medication will be given if necessary.
- **Infection –** Increased risk of intra-abdominal infection (peritonitis).
- **Delayed healing**
- **Lethargy/tiredness**
- **Diarrhoea**
- **Mucositis/stomatitis**
- **Allergic reactions (oxaliplatin)**
- **Neuropathy (oxaliplatin)**
- **Coronary artery spasm (fluorouracil)**
- **Severe 5-FU toxicity due to DPD deficiency (see above)**

A full list of expected toxicities can be viewed at the Electronic Medicines Compendium

Overview of treatment programme

Consider dose capping chemotherapy at BSA 2.2m^2.

Treatment is administered in surgical theatres:

<table>
<tr><th>Time</th><th>Drug</th><th>Dose</th><th>Route</th><th>Diluent</th><th>Administration</th></tr>
<tr><td colspan="6">Day 1</td></tr>
<tr><td>T=0</td><td>Calcium folinate</td><td>50mg (Flat dose)</td><td>IV bolus</td><td>-</td><td rowspan="2">To be administered 60 minutes before intraperitoneal chemotherapy.</td></tr>
<tr><td>T=0 mins</td><td>Fluorouracil*</td><td>400mg/m^2</td><td>IV infusion (over 60 minutes)</td><td>250mL sodium chloride 0.9%</td></tr>
<tr><td>T=60 mins</td><td>Oxaliplatin</td><td>368mg/m^2</td><td>Intraperitoneal (Perfused for 30 minutes)</td><td>-</td><td>For heated INTRAPERITONEAL perfusion with the carrier fluid. Dose to be prepared in an empty 5% Glucose bag.</td></tr>
<tr><td colspan="6">Once only treatment
• *The fluorouracil infusion is flushed through with 50mL sodium chloride 0.9%.
• Intraperitoneal oxaliplatin is mixed in 2 L/m^2 of 1.5% dextrose DIANEAL® PD4 peritoneal dialysis solution perfused for 30 minutes at intraperitoneal temperature >42°C using closed abdomen technique and hyperthermia pump, flow rate 1.2L/min.</td></tr>
</table>

Extravasation:

Fluorouracil is an inflammatant

Refer to the local Extravasation Guidelines

Additional medication

For most patients this regimen has low/moderate emetogenicity.

Checklist of initial investigations and work-up prior to start of treatment

Note that patients will be reviewed in pre-op clinic by anaesthetic team who will undertake a full medical history and physiological assessment. Further investigations may be requested at the discretion of the anaesthetist and oncology team.

- ☐ *Staging CT thorax/abdo/pelvis*
- ☐ *Medical history*
- ☐ *Physical assessment*
- ☐ *FBC, U&Es, LFTs, calculate creatinine clearance (CrCL)*
- ☐ *Tumour markers: CEA*
- ☐ *Ensure up to date height and weight are recorded*
- ☐ *Prescribe chemotherapy*

Dose modifications

HIPEC is a once only course, so cumulative adverse effects are not usually a concern. Any dose adjustments are at the discretion of the prescribing consultant oncologist.

**Haematological**			
Neutrophils		*Plt*	*Action*
≥*1.5*	***and***	≥*100*	*Go ahead with chemotherapy*
<1.5	**and/or**	<100	Consultant decision

**Renal impairment**	
Creatinine Clearance (ml/min)	*Action*
≥50	Full dose
<50	Consultant decision

**Hepatic impairment**
Consultant decision.

Post treatment follow-up

- Patient will be followed up by the surgical team as an outpatient following their discharge from the hospital and adequate recovery time.
- Oncological follow up will be with the patient's local team.

Change log

Date	Version no	Author	Changes
Feb 2019	1.0	Joanne Collins	-
March 2022	1.1	Joanne Collins	Regimen title updated.<br><br>DPYD testing information included.<br><br>HIPEC technique used is a closed technique, rather than open.<br><br>Renal dosing- Consultant decision where CrCl is <50ml/min.<br><br>References updated.

References

1. Chua TC, Moran BJ, Sugarbaker PH, et al. Early- and long-term outcome data of patients with pseudomyxoma peritonei from appendiceal origin treated by a strategy of cytoreductive surgery and hyperthermic intraperitoneal chemotherapy. J Clin Oncol 2012;30(20):2449–56.

2. Verwaal VJ, Bruin S, Boot H, van Slooten G, van Tinteren H. 8-year follow-up of randomized trial: cytoreduction and hyperthermic intraperitoneal chemotherapy versus systemic chemotherapy in patients with peritoneal carcinomatosis of colorectal cancer. Ann Surg Oncol 2008;15(9):2426–32.

3. Elias D, Gilly F, Boutitie F, et al. Peritoneal colorectal carcinomatosis treated with surgery and perioperative intraperitoneal chemotherapy: retrospective analysis of 523 patients from a multicentric French study. J Clin Oncol 2010;28(1):63–8.

4. Elias D, Lefevre JH, Chevalier J, et al. Complete cytoreductive surgery plus intraperitoneal chemohyperthermia with oxaliplatin for peritoneal carcinomatosis of colorectal origin. J Clin Oncol 2009;27(5):681–5.

5. The Christie NHS Foundation Trust. HIPEC - Guidance for the selection of IP chemotherapy agent

6. The Christie NHS Foundation Trust Peritoneal Tumour Service: HIPEC Protocol- oxaliplatin

7. The Christie NHS Foundation Trust Peritoneal Tumour Service: 102 Hyperthermic Intraperitoneal Chemotherapy (HIPEC) Information for patients (Sept 2014)

8. BC Cancer protocol. BCCA Protocol Summary for Hyperthermic Intraperitoneal Chemotherapy (HIPEC) for Patients with Peritoneal Carcinomatosis from Limited Advanced Colorectal and Appendiceal Carcinomas Using Oxaliplatin and Fluorouracil (5-FU) (revised Oct 2016). Available at: http://www.bccancer.bc.ca/chemotherapy-protocols-site/Documents/Gastrointestinal/GIHIPEC_Protocol.pdf

9. Kusamura S, Barretta F, Yonemura Y, Sugarbaker PH, Moran BJ, Levine EA, Goere D, Baratti D, Nizri E, Morris DL, Glehen O, Sardi A, Barrios P, Quénet F, Villeneuve L, Gómez-Portilla A, de Hingh I, Ceelen W, Pelz JOW, Piso P, González-Moreno S, Van Der Speeten K, Deraco M; Peritoneal Surface Oncology Group International (PSOGI) and the French National Registry of Rare Peritoneal Surface Malignancies (RENAPE). The Role of Hyperthermic Intraperitoneal Chemotherapy in Pseudomyxoma Peritonei After Cytoreductive Surgery. JAMA Surg. 2021 Mar 1;156(3): e206363.
